# Migration and proliferation drive the emergence of patterns in co-cultures of differentiating vascular progenitor cells

**DOI:** 10.3934/mbe.2024295

**Published:** 2024-08-01

**Authors:** Jose E. Zamora Alvarado, Kara E. McCloskey, Ajay Gopinathan

**Affiliations:** 1 School of Engineering, University of California Merced, Merced, CA 95343, USA; 2 Graduate Program in Materials and Biomaterials Science and Engineering, University of California Merced, Merced, CA 95343, USA; 3 Department of Physics, University of California Merced, Merced, CA 95343, USA

**Keywords:** computational model, stem cell differentiation, vascular development, patterning, endothelial cells, smooth muscle cells

## Abstract

Vascular cells self-organize into unique structures guided by cell proliferation, migration, and/or differentiation from neighboring cells, mechanical factors, and/or soluble signals. However, the relative contribution of each of these factors remains unclear. Our objective was to develop a computational model to explore the different factors affecting the emerging micropatterns in 2D. This was accomplished by developing a stochastic on-lattice population-based model starting with vascular progenitor cells with the potential to proliferate, migrate, and/or differentiate into either endothelial cells or smooth muscle cells. The simulation results yielded patterns that were qualitatively and quantitatively consistent with experimental observations. Our results suggested that post-differentiation cell migration and proliferation when balanced could generate between 30–70% of each cell type enabling the formation of vascular patterns. Moreover, the cell-to-cell sensing could enhance the robustness of this patterning. These findings computationally supported that 2D patterning is mechanistically similar to current microfluidic platforms that take advantage of the migration-directed self-assembly of mature endothelial and mural cells to generate perfusable 3D vasculature in permissible hydrogel environments and suggest that stem or progenitor cells may not be fully necessary components in many tissue formations like those formed by vasculogenesis.

## 1. Introduction

A major obstacle in the development of tissue engineered products for clinical applications is the challenge of generating perfusable vasculature, *in vitro*. This issue is amplified in both importance and scale when building larger or highly complex organs. While the decellularization of adult organs such as heart [1,2], lung [3–5], kidney [6,7], and liver [8,9] retains the highly branched vascular architecture, methods for cell seeding preformed matrix at physiologically-required cellular densities remains a challenge. Another promising approach to generating perfusable vasculature is seeds mature vascular cells into 3D hydrogels. Several groups have developed microfluidic platforms that permit the self-assembly of endothelial cells into perfusable vasculature [10–12] and direct anastomosis of that vasculature within the platforms microstructures. In these studies, co-cultures of human umbilical vein endothelial cells (HUVECs) and normal human lung fibroblasts (NHLFs) or pericytes (PCs) are seeded as single cells into fibrin or collagen gels [13–15]. Over 4–7 days, the migration and self-assembly of these cells leads to the formation of perfusable vessels. A third approach to generating perfusable vasculature uses developmental or differentiation methods enabling endothelial cell (EC) [16,17] and smooth muscle cell (SMC) [18–20] fate within organoid-like structures supplemented with growth factors, specialized cell culture mediums, and/or mechanical signaling [21] to guide the formation and development of perfusable vessels [22].

**Figure 1.**
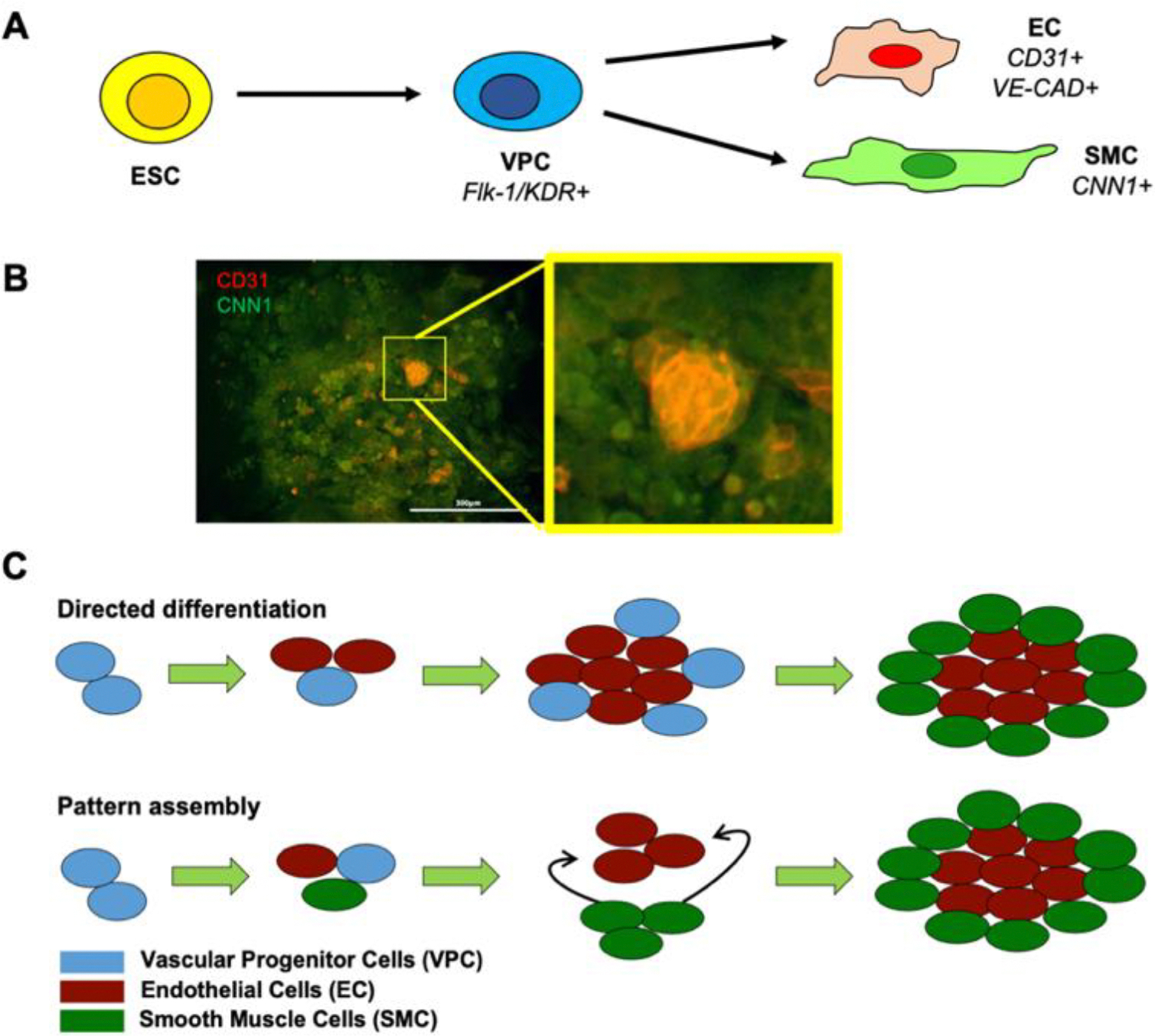
Experimental observations. A) Schematic of the differentiation process. Briefly, ESCs are differentiated into VPCs via stage specific induction medium. VPCs are then further differentiated into co-cultures of ECs and SMCs via stage 2 specific induction medium. B) Immunofluorescent images of VPC outgrowths at day 4 post-secondary induction, stained for CD31 (red) indicate cells committed to an EC fate, while CNN1+ cells (green) indicate SMC fate commitment. Magnified is an EC cluster (red) surrounded by SMCs (green). Scale bar is 300 μm. C) Schematic of possible mechanisms explaining emergence of micropatterns, either through directed differentiation where differentiation is spatially varying or via pattern assembly where post differentiation mechanisms, specifically migration and proliferation, are driving pattern formation.

Our laboratory’s chemically-defined differentiation protocols have proven highly effective in deriving mouse embryonic stem cells (ESCs) and human ESCs and induced pluripotent stem cells (iPSCs) [23–26] into vascular progenitor cells (VPC), ECs, and SMCs (Figure 1A). While obtaining purified differentiated vascular cells is an important first step, the ultimate goal is to simultaneously direct the emergence of vascular structures from differentiating cells as seen during normal development. Indeed, VPC outgrowths have been observed to self-organize into micropatterns (Figure 1B) with EC clusters loosely surrounded by SMCs [27]. However, in order to direct vascular patterning in 3D, one must understand and control the interplay of various processes involved in cell organization: Proliferation, migration, and differentiation. While the details of such strategies are highly system-dependent given the complexity and inter-connectedness of the processes, we sought to first understand the relative importance of these processes in patterning. For example, do cell patterns emerge from directed differentiation from neighbor cells or the migration and rearrangement of post-differentiated cells (Figure 1C)? Here, we examine the relative importance of physiologically relevant range of parameters using a computational model motivated and calibrated by our experimental cultures of mouse ESCs in 2D [27].

Several computational models exist for simulating the self-organization of multicellular tissues [28], including on-lattice and off-lattice models that account for cell adhesion, proliferation, and short or long range signaling. Depending on the specific question, different models exhibit distinct advantages. For example, vertex models are excellent for exploring adhesion, proliferation, cellular forces, and cellular geometries, but become computationally expensive with more cells [28]. Likewise, finite element models (FIE) have been used in modeling environments where cell geometries and cellular forces are important [29,30]. Many other models have explored cell differentiation [31,32], migration [33], proliferation [34], and combinations of these parameters [35–41]. Here, we chose an on-lattice, stochastic, population-based model that uses ordinary differential equations (ODE) to represent the spatial-temporal dynamics of cell densities (# of cells per lattice site) evolving through cell proliferation, migration, death, differentiation, and cell-to-cell signaling. Such an approach allows effective parametrization of the important processes, while also monitoring the spatial structure of the cellular populations over time. A coarse-grained approach also avoids replicating dynamics at the single cell level increasing computational efficiency in simulating the large cell numbers (10^4^–10^5^) observed in our experimental cell cultures.

Here, we simulated the time evolution of the system for a wide range of physiologically plausible parameters and output reporting the fractions and spatial distributions of the emerging vascular cells (ECs or SMCs). By analyzing simulations within physiologically relevant domains calibrated with experimental data, we extract practical information from each of the simulated parameter sweeps. The results suggest that the vascular-like spatial patterns (defined as EC clusters surrounded by SMCs) emerge when the fraction of differentiated ECs lies within a “zone of co-emergence” with well-balanced numbers of both cell types. Moreover, for physiologically relevant ranges of proliferation and migration, the distribution of simulated EC cluster diameters was consistent with experimental observations. For the parameter ranges relevant to our system, we found that the spatial distributions of different cells are more sensitive to differences in proliferation and migration rates between cell types compared with differences in intrinsic or induced differentiation rates. Consequently, it is the proliferation and migration rates that appear to be most responsible for the establishment of the observed micropatterns within our differentiating co-cultures. These results support the current self-assembly vasculogenesis observed in mature post-differentiation cell co-cultures when seeded withing hydrogels [10–12] and may aid the rational design of co-developing vasculature within 3D tissues.

## 2. Materials and methods

### 2.1. Embryonic stem cell culture

The mouse embryonic stem cell (mESC) lines used for these studies included mESC-R1 (ATCC). The mESC-R1 were cultured on 0.5% gelatin in serum-free medium containing Knockout Dulbecco’s Modified Eagle Medium (KO-DMEM; Invitrogen), 15% Knockout Serum Replacer (KSR; Invitrogen), 50 units/mL and 50 ug/mL of Penicillin-Streptomycin (Invitrogen), 1Χ Nonessential Amino Acids (Invitrogen), 2 mM L-glutamine (Invitrogen), 0.1 mM 2-mercaptoethanol (Calbiochem), 2000 Units/ml of leukemia inhibitory factor (LIF-ESGRO; Chemicon), and 10 ng/ml of bone morphogenetic protein-4 (BMP-4; R&D Systems). Full media changes occurred every other day and cells were passaged every four to five days and reseeded at a density of 10^4^ cell/cm^2^.

### 2.2. Embryonic stem cell differentiation

Mouse ESC-R1 (ATCC) were differentiated into EC and SMC using our laboratories two staged serum-free induction protocols [25]. Briefly, mESC were induced on 50 μg/mL fibronectin (Corning) coated plates (BD Biosciences) under our stage 1 induction medium containing alpha-MEM (Cellgro), 20% knockout serum replacement (ThermoFisher), 50 units/mL and 50 ug/mL of penicillin-streptomycin (ThermoFisher), 1Χ nonessential amino acids (ThermoFisher), 2 mM L-glutamine (ThermoFisher), 0.05 mM 2-mercaptoethanol (Calbiochem), 5 ng/mL BMP-4 (Peprotech), and 30 ng/mL of VEGF (Peprotech). After 2 days in culture, vascular progenitor cells (VPCs) were live stained for Flk-1 expression (1: 200, Biolegend) for 30 minutes, washed, and sorted by Fluorescence-activated cell sorting (BD FACS Aria III). Positive cells were replated on 50 μg/mL fibronectin for an additional 4 days under stage 2 specific differentiation medium consisting of 70% alpha-MEM (Mediatech), 30% DMEM (Invitrogen), 2Χ Nutridoma CS (Roche), 50 units/mL and 50 ug/mL of penicillin-streptomycin (Invitrogen), 1Χ nonessential amino acids (Invitrogen), 2 mM L-glutamine (Invitrogen), 0.05 mM 2-mercaptoethanol (Calbiochem), and supplemented with 50 ng/mL of basic fibroblast growth factor (bFGF) as previously optimized for mESC-R1 induction into ECs [25].

### 2.3. Immunofluorescent staining

Visualization of the cell micropatterning was conducted by immunofluorescent staining of VPCs outgrowths on day 4 post purification (Figure 1B). Briefly, cells were fixed with 4% paraformaldehyde (Tousimis) and permeabilized with 0.5% Triton X-100 (MP Biomedicals). Nonspecific binding was prevented using 1% bovine serum albumin (Sigma). Conjugated CD31 PE, EC stain (BD Biosciences) and primary antibody CNN1 (SMC stain, Sigma) were added and allowed to stain overnight at 4°C. Cells were rinsed before addition of secondary antibody, Alexa Flour 488 (Thermofisher), and DAPI. Cells were incubated for an additional hour before final rinse and imaging via fluorescence microscopy (Nikon TE2000-U). For F-actin staining we used an Alex Fluor 488 phalloidin stain (Invitrogen).

### 2.4. EC cluster diameter distribution

The diameters of EC clusters were calculated by a custom MATLAB script. Briefly, imported images, either experimentally obtained images of DAPI stained cells or computationally simulated clusters, were smoothed via *medfilt2* function, and then turned into binary (*imbinarize*) images via Otsu (*graythresh*) thresholding. An outline of the clusters was then reconstructed via subtraction of the *dilated* and *eroded* binary images. The *regionprops* function was then used to record the *Filled Area* of the clusters (Figure S1). Comparison between experimental and simulated clusters, was done by calculating an effective cluster diameter, D = 2*(*Filled Area* /π)^0.5^, for both conditions.

### 2.5. Modeling stem cell dynamics

**Figure 2.**
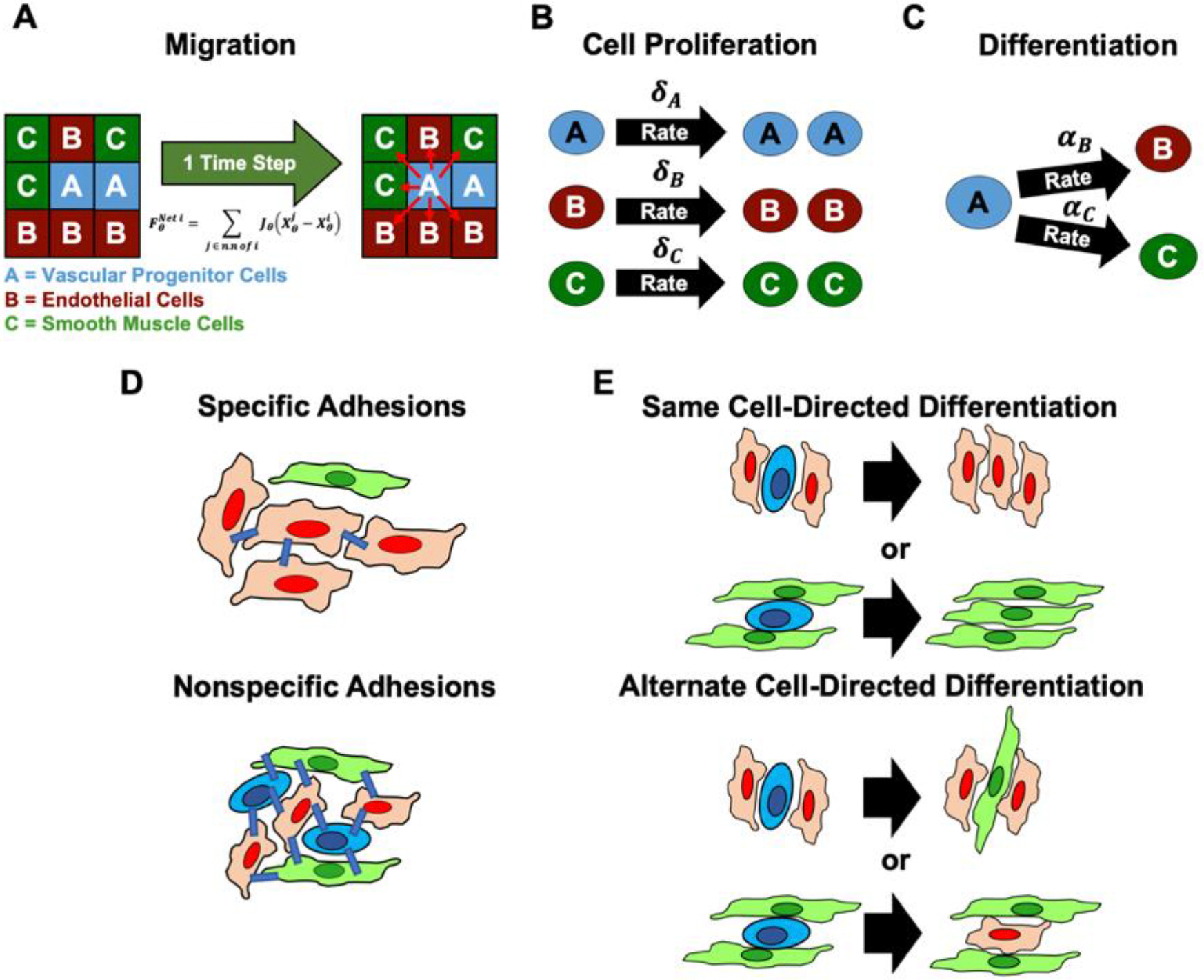
Modeled processes. A) cell migration is governed by the flux, F_θ_, of cells moving between lattice sites due to cell density gradients. The schematic illustrates how there is a net movement of VPCs into lattice sites that contain fewer VPCs. B) Cell proliferation, δ_θ_, is assumed to be symmetric where one cell type will always produce more of the same cell type at a specified rate. C) Cell differentiation, α_θ_, is assumed to be a nonreversible fate decision made by VPCs (A cells) being driven towards one of two possible mature cell types: ECs (B cells) and SMCs (C cells) at rates α_B_ and α_C_, respectably. D) We assume contact inhibition to regulate the proliferation and migration of the cells. One such mechanism would be mediated by specific adhesions, where cells only sense cells of the same identity. Alternatively, they can be mediated by nonspecific adhesions, where cells indiscriminately sense other cells. E) Paracrine signaling is similarly dependent on the local cellular microenvironment via biochemical cues that nudge VPCs towards a specific fate identity, EC or SMC, respectively. Here two possible mechanisms arise, same cell-directed differentiation where committed cells, ECs or SMCs, direct the differentiation of VPCs into the same cell identity, or alternate cell-directed differentiation where committed cells influence the differentiation of VPCs into the opposite cell type.

Our computational model considers the spatial and temporal distributions of three different cell types within a simulated square lattice. We denote by $X_{A},\;X_{B}$, and $X_{C}$ the normalized cell density (e.g. $X_{A}=N_{A}/N_{max}$, where $N_{A}$ is the number of A cells and $N_{max}$ is the minimum number of cells of all types put together allowed at each lattice site leading to X belonging to the interval [0, 1]) of VPCs, ECs and SMCs, respectively. Their dynamics are governed by the cell’s migration, their rates of proliferation, differentiation, and cell death (Figure 2A–C). In our model, each cell can migrate between neighboring lattice sites at a preset migration value, J_θ_ (here θ represents either A, B or C corresponding to cell identities of VPC, EC, and SMC, accordingly). Each cell type can also proliferate (double), and die at a specific rate, δ_θ_ and μ_θ_, respectively. Additionally, VPCs can differentiate into either ECs or SMCs at rates of α_B_ and α_C_, respectively. The cellular dynamics are then a combination of a cell’s migration, these rates, and stochastic noise, S_θ_, leading to a set of three ordinary differential equations (ODEs), Eqs (1)–(3), one for each of the three changing cell populations ($X_{A},\;X_{B}$, and $X_{C}$).

(1) $${\dot{X}}_{A}=\left(\left(\delta_{A}+S\right)(1-\sum_{\theta \;\in \;A,B,C}\;\left[X_{\theta }^{i}\right])-\mu_{A}\right)X_{A}-\left(\alpha_{B}+\alpha_{C}\right)X_{A}+F_{A}$$

(2) $$\dot{X_{B}}=\left(\left(\delta_{B}+S\right)(1-\sum_{\theta \;\in \;A,B,C}\;\;\left[X_{\theta }^{i}\right])-\mu_{B}\right)X_{B}+\alpha_{B}X_{A}+F_{B}$$

(3) $${\dot{X}}_{C}=\left(\left(\delta_{C}+S\right)(1-\sum_{\theta \;\in \;A,B,C}\;\;\left[X_{\theta }^{i}\right])-\mu_{C}\right)X_{C}+\alpha_{C}X_{A}+F_{C}$$

here, noise is captured by a stochastic addition to the combined rates of proliferation and death. The stochastic noise, S_θ_, is drawn from a normal distribution and is assumed, for simplicity, to be the same for all cell types. The factor of $\left(1-\sum_{\theta \in A,B,C}\;\left[X_{\theta }^{i}\right]\right)$ modulates the growth rate relative to available space and accounts for the carrying capacity of each lattice site i.e., the sum of the normalized cell densities cannot exceed 1 and the growth rate vanishes as the total density approaches 1. The last term in each equation accounts for the flux of cells, F_θ_, into a lattice site which depends on the cell’s migration rate, J_θ_. In the absence of any biases or interactions, these fluxes are proportional to the cell density gradient for a specific cell type, summed over all nearest neighbor sites, with a constant of proportionality reflecting the migration of the cell type Eq (4).

(4) $$F_{\theta }^{i}=\sum_{j\;\in \;n.n\;of\;i}\;J_{\theta }\left(X_{\theta }^{j}-X_{\theta }^{i}\right)$$

The range of physiologically relevant migration, proliferation, and differentiation rates we used were derived from various literature sources. For example, prior studies have reported that ECs and SMCs can have a maximum displacement distance of 18 and 44 μm over the course of an hour, respectively [33,42]. Additionally, proliferation rates for mouse ECs have been reported to have a minimum doubling time of 19 hrs [43], while 22 hrs has been reported for rat aortic vascular SMCs [44]. Last, the rate of differentiation can be considered as the length of time for specific EC or SMC marker expression. Some studies have reported initial EC marker expression at as early as 24 hrs post induction [45] and 36 hrs for SMC marker expression [46], while most studies do not see differentiation makers emerging before 5–7 days [23] from mouse stem cells and 10 to 20 days from human stem cells [47]. Using these physiologically relevant values for our parameters (see Table 1; Supplementary video V1), our simulations were evolved in time using the Euler (first order Runge-Kutta) method implemented in Python [48]. Each cell’s population density, at each lattice site, is tracked over time (recorded every 1hr in time) and can be reconstructed to create a 2D spatial representation of the evolving cell populations using MATLAB [49] (Supplementary video V2 and Figure S2A–C).

### 2.6. Contact inhibition and paracrine signaling

Under the simplest assumption, the various rates (in Table 1) for specific cell types are fixed. However, these rates can also be modulated by different types of external cell interactions, such as contact inhibition and paracrine signaling [25,50]. To explore how these external processes affect patterning, we accounted for their promotion or suppression of the corresponding rate constants for migration, proliferation, and differentiation.

To model the effects of contact inhibition, we assumed that a cell’s migration rate would simply decrease linearly with the number of cells in its immediate neighborhood (summed over the site and all nearest neighbor sites) [51]. Within this scheme, we examined two distinct possibilities, specific and nonspecific cell adhesions, as possible regulators (Figure 2D). For specific homotypic cell adhesions, we assumed that cells can only sense and interact with other cells of the same cell identity, leading to a modified migration rate,

(5) $$J_{\theta }=J_{\theta }^{o}\left(1-\left(\frac{x_{\theta }^{i}+\sum_{j\;\in \;n.n\;of\;i}\;\;X_{\theta }^{j}}{z}\right)\right)$$

where i,j are site indices and z is the coordination number of nearest neighbor lattice sites plus the current site (z = 9). The migration rate thus decreases from a maximum for isolated cells to zero when the cell density of the same type is maximal. For nonspecific cell adhesions, we assume cells can sense all other cells in their local neighborhood (leading to Eq (6)).

(6) $$J_{\theta }=J_{\theta }^{o}\left(1-\left(\frac{\Sigma_{\theta \;\in \;A,B,C}[X_{\theta }^{i}+\sum_{j\;\in \;n.n\;of\;i}\;\;X_{\theta }^{j}]}{z}\right)\right)$$

here, the migration rate decreases from a maximum for isolated cells to zero when the overall cell density for all cells is maximal. Combinations with different cell types employing different sensing mechanisms were also explored. For example, ECs could be modeled to nonspecifically sense their neighboring cells, leading to greater contact inhibition, while SMCs could be assigned to specifically sense other SMCs (consistent with homotypic sensing). It is to be noted that patterning arising from variations in adhesion is consistent with the differential adhesion hypothesis [52], which states that cells with strong attractions will cluster closely together while those with weaker attractions will surround them. Here we account for the migration of cells being differentially affected by differences in cell adhesion dynamics mediated by specific vs nonspecific sensing. An example of a strong (homotypic) cell-cell adhesion would be vascular endothelial cadherins (VE-cadherins) that hold ECs together [53] with a binding force between 35–55 pN [54], while E-cadherin which can binds ECs to SMCs is an example of a nonspecific cell adhesion [55] with a binding force between 32–48 pN [56].

A similar approach was implemented for modifying the proliferation rates based on homotypic and nonspecific cell adhesion. We assumed that proliferation would linearly decrease as the number of neighboring cells increased, in accordance with evidence demonstrating that the mitotic rate may be arrested when a critical cell confluency is achieved. This type of contact inhibition emerges from an increase in mechanical interactions resulting from the loss of available space, thus constraining cellular dynamics including cell division [41]. It is also possible for arrest to occur in a specific manner such as being mediated by the EC specific Notch signaling pathway that inhibits tip-EC proliferation during angiogenesis [57]. Therefore, we modified the proliferation rates appropriately to account for two different sensing mechanisms: 1) Specific, homotypic, cell sensing:

(7) $$\delta_{\theta }=\delta_{\theta }^{o}\left(1-\left(\frac{x_{\theta }^{i}+\sum_{j\;\in \;n.n\;of\;i}\;X_{\theta }^{j}}{z}\right)\right)$$

and 2) nonspecific cell sensing:

(8) $$\delta_{\theta }=\delta_{\theta }^{o}\left(1-\left(\frac{\Sigma_{\theta \;\in \;A,B,C}[X_{\theta }^{i}+\sum_{j\;\in \;n.n\;of\;i}\;X_{\theta }^{j}]}{z}\right)\right)$$

These proliferation rate dependencies are analogous to Eqs (5) and (6) for the migration rates, with a coordination number (z) equal to 9.

For differentiation, we examined how chemical signals and growth factors produced by the cells influence VPC fate decisions, modeling vascular paracrine signaling [58]. Such signaling is observed in EC and SMC recruitment during early vascular development, with ECs secreting platelet-derived growth factor-b when recruiting SMCs [53]. We explored two possible mechanisms for guiding VPC differentiation into ECs and SMCs. The first mechanism, designated same cell-directed differentiation, involves a committed cell, EC or SMC, inducing a neighboring uncommitted VPC to differentiate into a cell of the same identity. The second mechanism, designated alternate cell-directed differentiation, assumes a committed cell, such as an EC, influencing a neighboring VPC to instead differentiate into the alternative cell type, in this example inducing it into a SMC (Figure 2E). For simplicity we assumed an isotropic distribution of paracrine signals for both cell types, where the diffusion range is equivalent to one lattice unit. Given that our lattice size is 79 μm (see next section), this is a reasonable upper bound for diffusive signal propagation. The modified differentiation rate is then given by

(9) $$\alpha_{\theta }=\alpha_{\theta }^{o}\left(1\pm \beta \left(\frac{X_{\theta^{\prime }}^{i}+\sum_{j\;\in \;n.n\;of\;i}\;X_{\theta^{\prime }}^{j}}{z}\right)\right)$$

where the choice of EC or SMC, for θ and $\theta^{’}$, determines the type of cell-directed differentiation mechanism (Figure S3) and β represents the strength of the signaling from cells in neighboring lattice sites.

### 2.7. Simulations

At time 0 (t = 0), a fraction of our 50×50 lattice is randomly seeded with VPC population densities, XA, with values taken from a normal distribution between [0, 1]. Here each unique lattice site supports up to 10 cells and has a lattice unit size of 79 μm (Figure S2A). This choice of lattice size was motivated by the average cell size of our VPCs, measured to be 25 ± 7 μm across (Supplementary video S1, Figure S2D–E), whereby each lattice site is significantly larger than 1 cell diameter, so that our population-based approach remains valid, and yet small enough to visualize the population driven micropattern features consisting of mature cell clusters with effective diameters of 340 ± 110 μm (Figure S1G). The fraction of sites seeded was chosen such that the seeded density for the array was consistent with experimental cell seeding densities of VPCs (of 10^4^ cells/cm^2^) [25]. A migration constant value was derived from the average distance traveled by randomly migrating human umbilical vein ECs, of about 14 μm over an hour [33]. Taking it to be a random walk process, we can compute an effective diffusion constant, D, from mean-squared displacement (<r^2^>~(14 μm)^2^) and the time taken (t), as <r^2^>/4t = D. Non-dimensionalizing D using the lattice site dimensions (79 μm) as the unit of length and 1hr as the unit of time, we obtained a corresponding dimensionless migration constant of J_θ_ = 0.0079. Additionally, we chose to fix the baseline (unmodified) values for proliferation and differentiation rates within physiological ranges (Table 1). These rates were calculated from the typical times associated with the given processes. We set the rate of proliferation for all cells to 40 hrs [43,44,59], corresponding to a dimensionless simulation value of δ_θ_ = 0.025. The differentiation rate was set to 62.5 hrs [27] thus falling between days 2–3 of post stage 2 differentiation, corresponding to a simulation value of α_θ_ = 0.016. Once the simulation is initialized, the three first order ODEs, Eqs (1)–(3), are evolved in time using the Runge-Kutta method, and X_θ_ values are recorded at every lattice site at 1-hr intervals for the duration of the simulation (Figure S2B,C). At t = 96 hrs, corresponding to experimental data at day 4, the recorded information is quantified and analyzed. We recorded the populations size for each cell type, their spatial distribution, and the fraction of ECs and SMCs at t = 96 hrs, under each specific condition. Supplementary video V2, shows a simulation evolving in time, for a particular set of parameter values. We conducted parameter sweeps over regions of parameter space that include experimentally relevant and physiologically plausible (see Table 1) values for each set of parameters. Simulations were run a total of 10 times for each set of parameters for statistical analysis.

## 3. Results

Using our stochastic on-lattice population-based model, we will explore the role that migration, proliferation, and differentiation rates have on the type of patterns that emerge from VPC (A cells) as they differentiate into ECs (B cells) and SMCs (C cells). We will also explore the cluster dynamics of a single EC cluster under various migration rates, and end by simulating different types of cell-to-cell signaling such as contact-inhibition and paracrine signaling.

### 3.1. Role of cell migration

**Figure 3.**
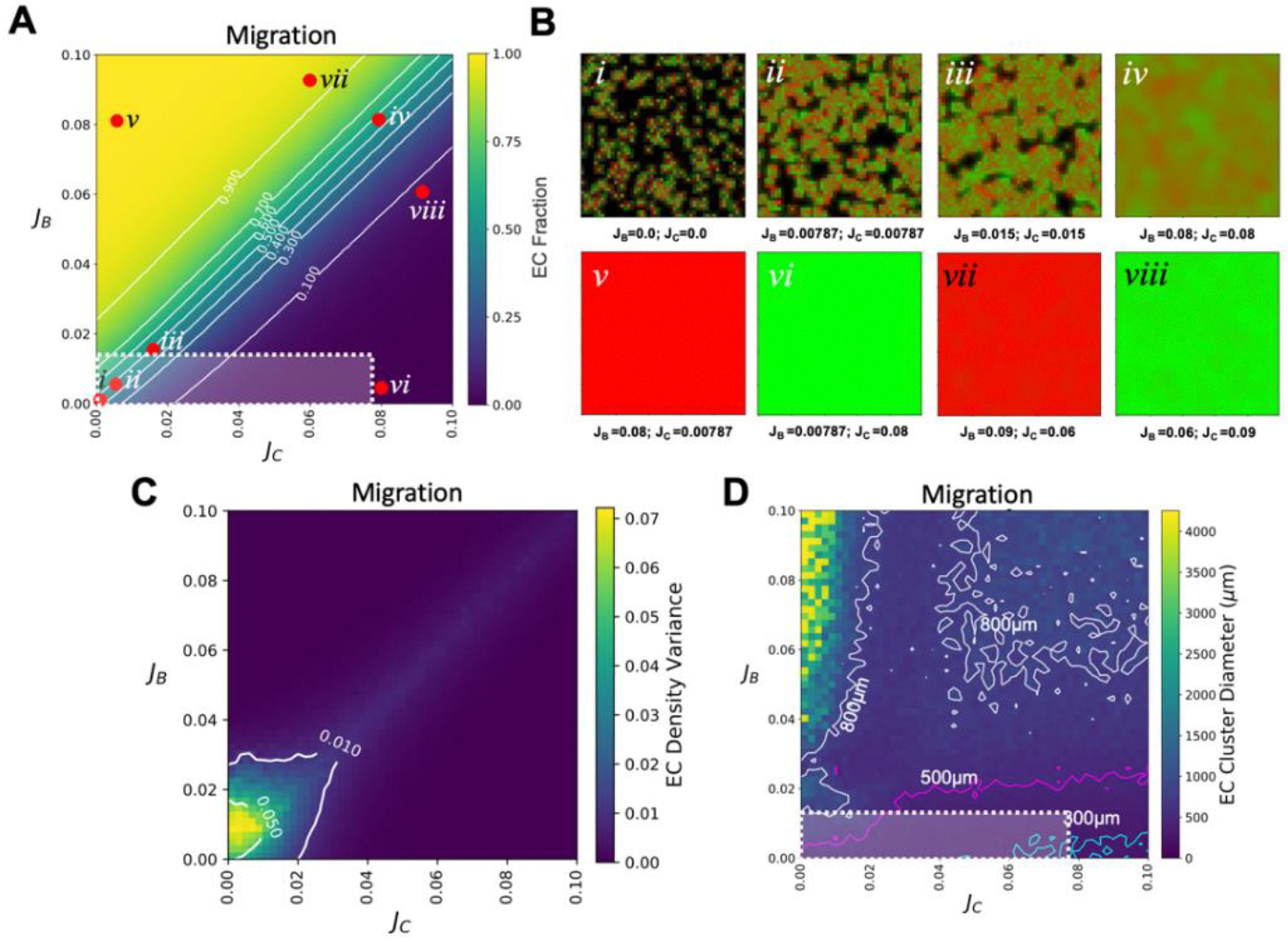
Effect migration has on micropatterning. A) Parameter sweep for migration (in the absence of sensing) was explored by varying J_B_ and J_C_ values while holding differentiation and proliferation rates constant. Shown here are the EC (B cell) fraction. Note: The presence of the zone of co-emergence, a region of parameter space where micropatterning is observed (defined by the area between 0.3~0.7 of EC fraction). B) Examining different combinations of J_B_ and J_C_ reveals the type of micropattern that develops after 96 hrs. Shown here, are the resulting pattern from within the zone of co-emergence (*i, ii, iii,* and *iv*), and from outside the zone of co-emergence where the faster cell type dominates (*v, vi, vii,* and *viii*). C) EC density variance plots show where distinct EC clusters emerge as migration is varied. D) Parameter sweeps of mean effective EC cluster diameter distributions: With contours 300 μm-cyan, 500 μm-magenta, and 800 μm-white. White squares in A) and D) denote the physiologically relevant region of the parameter space. Defined, for migration, as being the maximum speed that a single EC and SMC can migrate/move over a given amount of time. For migration those bound are between 0–0.013 for J_B_ and 0–0.078 for J_C_ corresponding to an upper limit representing the maximum reported single cell velocity, 18 μm/hr for ECs and 44 μm/hr for SMCs.

We first explored the role that cell migration, for EC (B cells) and SMC (C cells), has on the emerging micropattern. Our parameter sweep explored physiologically relevant migration values, between 0–18 μm^2^/hr for ECs and 0–44 μm^2^/hr for SMCs [33,42] corresponding to in-silico values of J_B_ = 0–0.013 and J_C_ = 0–0.078, respectively. We varied the ECs’ (J_B_) and SMCs’ (J_C_) migration rates while holding the VPC’s (A cells) migration (0.0079) constant and setting all other parameters, such as differentiation (0.016), proliferation (0.025), and noise (0.01) to be the same for all three cell types (see Table 1). The fractions of ECs at t = 96 hrs were then plotted over this parameter space (Figure 3A). We verified that roughly equal migration values for J_B_ and J_C_, generated relatively equal population densities of ECs and SMCs (EC fraction~0.5). The spatial distribution of cells in this regime also revealed micropatterning, defined as spatially separated regions dominated by one cell type or the other (Figure 3B i–iv). We observed that the micropatterning persists but becomes less distinct as the overall migration rates increased (compare Figure 3B i to iv). Moreover, micropatterning was observed over a broad range of EC fractions, roughly between 0.3 to 0.7, demarcating a “zone of co-emergence” (Figure 3A).

This zone of co-emergence can be quantified by cell type asymmetry (Figure S4A), where asymmetry is defined as (B_T_ − C_T_)^2^/(B_T_ + C_T_)^2^ where B_T_ and C_T_ are the total number of ECs and SMCs in the simulated space at t = 96 hrs. Here values of asymmetry close to 0 indicate similar population densities of ECs to SMC, while values closer to 1 denote the dominance of one cell type. The corresponding zone of co-emergence can be distinctly observed at asymmetry values up to 0.3 (Figure S4A). Interestingly, in our laboratory’s experimental studies on these same differentiating co-cultures of VPCs [27], we reported EC fractions to be 0.30 at day 3 and 0.25 at day 7 with distinct patterns being observed at day 7. The standard deviations from the stochastic effects introduced are averaged over 10 simulations and were observed to be insensitive to repeated runs, with any variance localized within the zone of co-emergence (Figure S4B). Next, we looked at the cases where one cell type migrated an order of magnitude faster than the other cell type. Not surprisingly, we saw that the faster cell type dominates and outcompetes the slower cell type for space (Figure 3B, v and vi). However, even a more modest difference of about 50% in the migration rates allowed the faster cell type to dominate (Figure 3B vii and viii). In fact, upon further investigation, the zone of co-emergence, is highly sensitive to differences in migration rates and with differences as small as roughly half a cell size per hour (~4.5 μm/hr), we observe a significant dominance of the faster cells (Figure S5).

The region of parameter space within the zone of co-emergence where micropatterning occurs was next explored. To do this, we quantified the spatial separation between the cell types, by measuring EC density variance, Avg[(X_B_-X_Avg(B)_)^2^], where X_B_ is the density of ECs at a given lattice site and X_Avg(B)_ is the average density of ECs taken over the entire simulated space at t = 96 hr (Figure 3C). Here, values close to 0 suggest no distinct separation of the ECs from the SMCs, while positive values indicate varying degrees of separation. As expected, we see a faint positive streak whose position mirrors the zone of co-emergence from the EC fraction plots (Figure 3A). This positive streak approaches 0 with increasing migration values for both ECs and SMCs and aligns with the simulated micropattern mixing observed under high migration values (Figure 3B.iv). Interestingly, micropatterns at lower migration rates, and where cell type mixing is less, were observed to have greater cell-type separation and therefore were more distinct (Figure 3C).

Finally, to quantitatively compare the simulated micropattern predictions with experimentally observed micropatterns, we measured the distributions of EC cluster diameters (see Methods). We found that the majority of the simulated EC cluster diameters, within the physiological range of migration, had effective diameters between 300–800 μm (Figures 3D and S4D). Here, the boundary for the physiological range, J_B_ = 0–0.013 and J_C_ = 0–0.078, outlined by white dashed lines, is set by the maximum reported migration values for ECs [33] and SMCs [42] (Figure 3D). The experimentally observed EC cluster diameters were found to be between 340 ± 110 μm (Figure S1) indicating that our simulations do account for the experimentally observed patterning within the explored physiological range. Last, the simulated EC cluster distribution diameters within the corresponding zone of co-emergence increased as migration increased for both cell types. At very high migration rates, well outside the experimental range, clusters with effective diameters exceeding 800 μm emerged (Figures 3D and S4D), though patterning became less distinct (as seen in Figure 3B*iv*).

### 3.2. Role of cell proliferation

**Figure 4.**
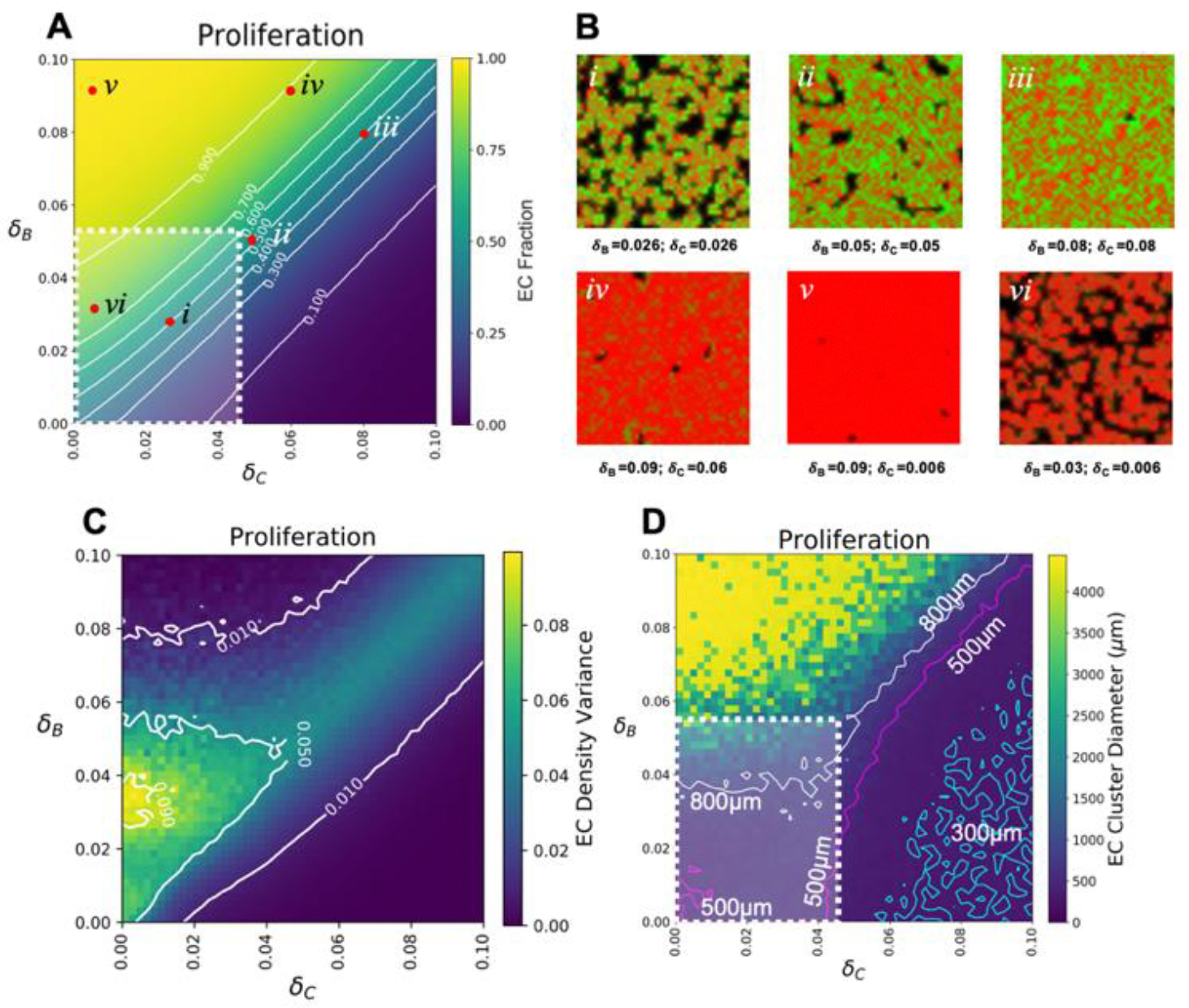
Effect proliferation has on micropatterning. A) EC (B cell) fraction parameter sweep for varying proliferation rates, in the absence of sensing, while holding differentiation and migration constant. B) Different combinations of δ_B_ and δ_C_ reveal the types of micropattern that develops after a 96 hr simulation. Shown here are micropatterns that emerge along the zone of co-emergence *i-iii*) and those outside the zone *iv-vi*). C) EC Density variance plots for distinct EC clusters as a function of varying proliferation rates. Positive values indicate varying degrees of EC cluster separation as defined by a greater EC density than the total averaged EC density per condition. D) Parameter sweeps of mean effective EC cluster diameter distributions. Contours here denote the relative cluster diameters that result as the rates are varied: Counters denote 300 μm-cyan, 500 μm-magenta, and 800 μm-white. Outlined in white in A) and D) is the physiological region defined, for proliferation, as the area bound between 0–0.055 for δ_B_ and 0–0.045 for δ_C_. The upper limit of which correspond to a cell’s minimum doubling time, here 18 hrs for ECs and 22 hrs for SMCs.

Next, we performed a parameter sweep of proliferation rates in the absence of any sensing mechanisms (Figure 4A). Proliferation rates were varied for ECs (B cells, δ_B_) and SMCs (C cells, δ_C_), while the proliferation rate for VPCs (A cells, δ_A_) and differentiation and migration rates for all cells were fixed at their experimentally relevant values (see Table 1). Reported doubling times include 19 hrs for mouse ECs [43] and 22 hrs for rat vascular SMCs [44]. The parameter sweeps therefore explored physiologically reasonable proliferation rates ranging from no cell division (δ_θ_ = 0) to divisions occurring as fast as every 10 hrs (δ_θ_ = 0.1). This region in parameter space is demarcated by white dashed lines in Figure 4A. Similar to the phase behavior with varying cell migration, the parameter sweeps here revealed that roughly equal rates of proliferation for ECs, δ_B_, and SMCs, δ_C_, lead to the formation of a zone of co-emergence within which micropatterning develops (Figure 4B i–iii). At the center of the zone, where δ_B_ and δ_C_ are both 0.05, we found highly distinct micropatterning emerge (Figure 3B ii). As expected, co-culture patterning largely developed for proliferation rates within the confines of the zone of co-emergence while parameter values outside the zone led to the faster growing cell dominating (Figures 4B iv–vi and S4E).

We then looked at the EC density variance plots which shows regions where distinct micropatterns of ECs emerged as a function of proliferation rates (Figure 4C). It was observed that varying the proliferation rates allowed for a larger span of conditions where cell-type separation, defined as having a finite EC cluster density, and thus micropatterning would emerge. Similar to the case with migration, we observe enhanced separation within the zone of co-emergence (Figure 4B i–iii). Interestingly, we observed that some separation persisted beyond the zone of co-emergence, where conditions favored EC proliferation rates over SMC rates (Figure 4C). In these instances, it was observed that the emerging EC clusters were predominately islands of various sizes, and while SMCs were present, they did not in fact surround EC clusters. Additionally, a region of high phase separation was observed at low SMC proliferation rates, δ_C_, and EC proliferation rates of 33 hr, δ_B_ = 0.03. This region, which again is outside the zone of co-emergence, consisted largely of only ECs clusters (Figure 4B vi, Figure 4C yellow region).

Last, we measured the distribution of EC cluster diameters and found that they consisted of clusters whose effective diameters were roughly between 500–800 μm, within the physiological range (white dotted lines, Figure 4D), with a broad distribution with standard deviation of 300–800 μm (Figure S4H). Finally, as proliferation rates increased for both ECs and SMCs, a narrow region of parameter space, with EC clusters between 500 and 800 μm, emerged and mirrored the position of the zone of co-emergence (Figure 4D).

### 3.3. Role of differentiation

**Figure 5.**
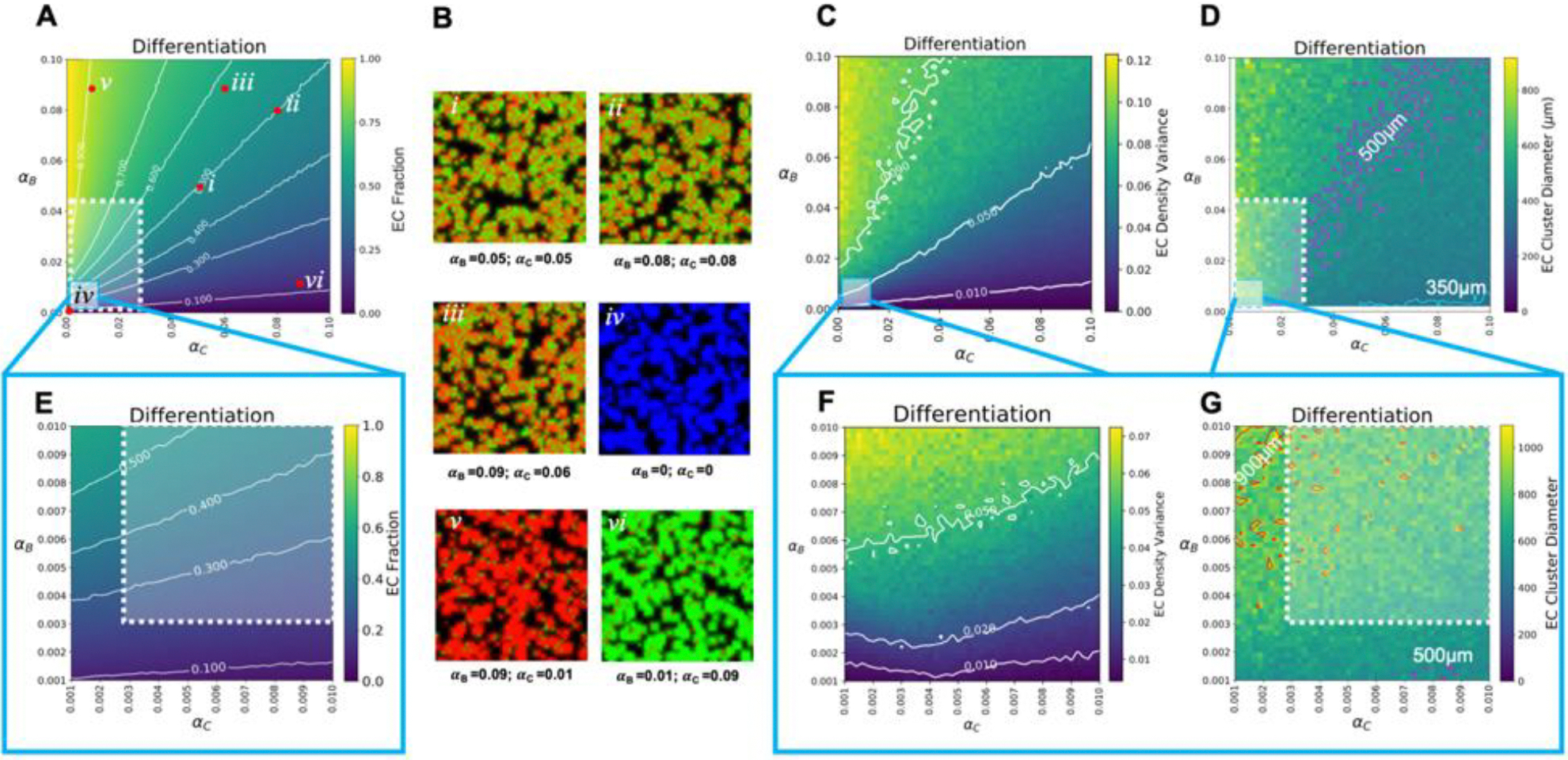
Effect differentiation has on micropatterning. A) EC (B cell) fraction parameter sweep for varying differentiation rates, in the absence of cell signaling, while holding proliferation and migration rates constant. B) Different combinations of α_B_ and α_C_ reveal the types of micropattern that develops after a 96 hr simulation. Shown here are micropatterns that emerge within what we consider the region of co-emergence *i-iv*) and those outside the region *v-vi*). C) EC Density variance plots for distinct EC clusters as a function of varying differentiation rates. D) Parameter sweeps of mean effective EC cluster diameter distributions. Contours here denote the relative cluster diameters that result as the rates are varied: Contours denote 350 μm-cyan, 500 μm-magenta. Further exploration of the lower bounds for differentiation are shown in E–G) corresponding to E) EC Fraction, F) EC density variance, and G) EC cluster diameter distribution. Here the white boundaries indicate the physiologically relevant domain for differentiation. The bounds are between 0.003–0.042 for ECs and 0.0028–0.028 for SMC corresponding to a differentiation rate of 24 hrs to 14 days for ECs and 36 hrs to 15 days for SMCs.

Next, we explored the role that differentiation plays in the emergence of micropatterns in the absence of any sensing or signaling. The parameter sweeps were conducted by varying the differentiation rates for both ECs (B cells, α_B_) and SMCs (C cells, α_C_) ranging between no differentiation (α_θ_ = 0) and a differentiation time of 10hrs (α_θ_ = 0.1), while holding the migration and proliferation rates constant and at experimentally relevant values (See Table 1, Figure 5A). Narrowing down the physiologically relevant parameter space for differentiation proved to be a difficult task given the variance under different conditions (e.g., chemical, mechanical, and/or contact mediated). Therefore, we define the physiologically relevant differentiation rate range as that between the first/early marker expression to the time point where the given marker expression peaks, indicating a mature phenotype. For mouse ECs it has been reported that VE-cadherin, a known EC marker, expression can be observed as early as 24 hrs post induction [45], corresponding an upper limit of α_B_ = 0.042. Additionally in one of our previous study, we showed that VE-cadherin peaks in differentiating mouse cells around day 14 post induction [25], corresponding a lower limit of α_B_ = 0.003. For mouse smooth muscle cells, early marker expression of α−smooth muscle actin (α-SMA) has been reported at 36 hrs post induction [46], thus establishing an upper limit of α_C_ = 0.028. Furthermore, peak α-SMA expression is reported to arise on day 15 [60], corresponding to a lower limit of α_C_ = 0.0028.

Upon analysis of the EC fraction parameter sweep, we noticed the absence of a sharply defined zone of co-emergence (Figure 5A), defined as EC fractions roughly between 0.3 to 0.7, and instead saw a large fanned out area of parameter space exhibiting micropattern formation (Figure 5B i–iii). As expected, when both differentiation rates, α_B_ and α_C_, are zero only VPC (A cells) are present (Figure 5B iv). Additionally, a loss of patterning only occurred when the differentiation rate of one cell type exceeded the other cell type by more than a factor of 2–3 (Figure 5B v–vi). This is also reflected in the asymmetry plots (Figure S4I) and in the EC density variance plots (Figure 5C), where an increasing gradient of EC density variance values is observed rather than the localized regions of cell-type separation observed in the migration and proliferation EC density plots. This gradient highlights the lack of impact that differentiation rates ultimately have on pattern formation as long as the differentiation rates of EC/SMC cell types are within a factor of 2–3 of each other. Last, the EC cluster diameter distribution was also observed to be fairly constant with cluster diameters ranging from 350 to 800 μm for the total explored parameter space (Figure 5D). A similarly broad range was recorded for the standard deviation, 10 to 500 μm (Figure S4L). To further explore the lower limits of the differentiation rates we repeated our simulations focusing on these longer differentiation periods. Here the range of interest was between 0.001 to 0.01, corresponding to differentiation rates of 1000 hrs [~42 days] to 100 hrs [~4 day]. Unsurprisingly, we did not see any defining differences at these lower bounds (Figure 5E–G).

### 3.4. Dynamics of cell clusters

**Figure 6.**
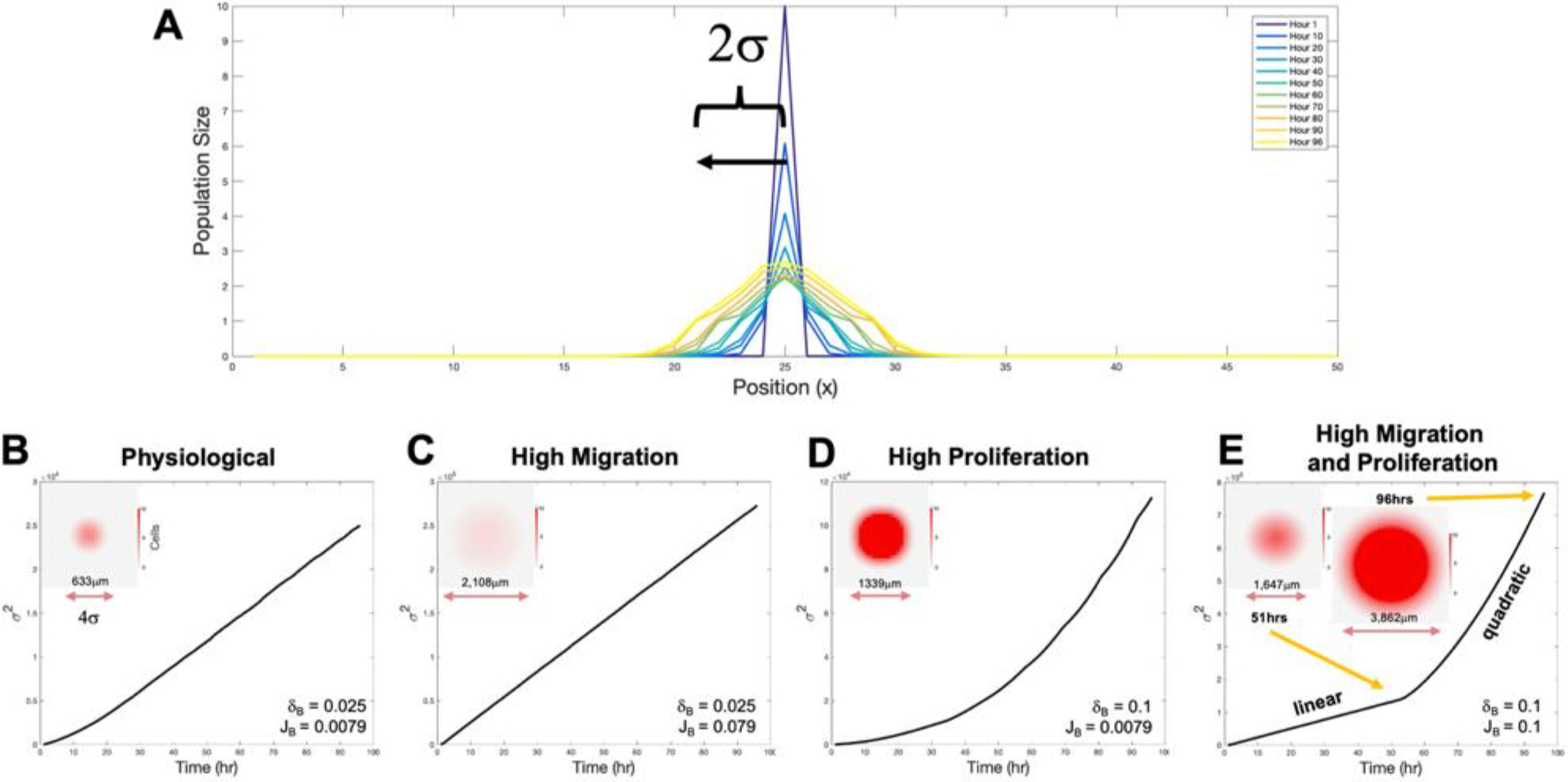
Single EC cluster growth dynamics. A) Plot of EC cluster growth over time along the x axis. Here the spread is defined as 2 s where s is the directional growth and diffusion along one of the x axis directions viewed from the center of the growing EC cluster. MSD plots over time for B) physiological conditions, C) under high migration rates, D) under high proliferation rates, and E) under both high migration and high proliferation rates. Inset here are the different EC cluster morphologies that emerge as a result from these rates with diameter size. Inset scalebar goes from 0 to 10.

So far, the results suggest that it is the post-differentiation migration and well-balanced proliferation of the cells that lead to cell patterning. A defining feature of this micropatterning that emerges within the zone of co-emergence is the size of the EC clusters. To understand the effect that migration and proliferation have on a cluster’s growth and size, we focused on the growth dynamics of a single EC cluster. We initialized the simulation with 10 cells at the center of the simulation site. As the ECs migrate and proliferate into neighboring lattice sites, the extent of their spreading can be calculated and visualized over time. To do this, we constructed a one-dimensional visualization of the EC spread over time by plotting the EC population along the x-axis as a function of time (Figure 6A). For physiological rates, i.e., when J_B_ = 0.0079 and δ_B_ = 0.025, we observed a rapid exodus from the center lattice site into the vacant neighboring lattice sites over time. Additionally, we observed two different regions emerge in the growing EC cluster. First, a leading diffusive front consisting of fractional EC densities, characterized by densities between 0.1 and 1 cells per lattice site. Second, a growing/expanding inner core region in the center, characterized by the presence of 2 or more ECs per lattice site (Figure 6B inset).

To quantify the size of the cluster, we then calculated the radius of gyration of the cluster in the x-direction, defined as $\sigma^{2}=\sum \left({x_{i}}^{2}*P_{EC}\left(x_{i}\right)\right)$ where x_i_ is the lattice position and P_EC_(x_i_) is the normalized EC population density along the x-axis such that P_EC_(x_i_) is equal to $X_{B}\left(x_{i}\right)\;/\;\sum \left(X_{B}\left(x_{i}\right)\right)$. The radius of gyration was then plotted over time (Figure 6B–E) followed by a linear and/or quadratic fit, representing diffusive and ballistic regimes respectively, with corresponding R^2^ values Tables S1–S4). Here, we considered four different cases (i) physiological migration and proliferation rates (where J_B_ = 0.0079 and d_B_ = 0.025), (ii) high migration (J_B_ = 0.079) with a physiological proliferation rate (d_B_ = 0.025), (iii) physiological migration (J_B_ = 0.0079) with a high proliferation rate (d_B_ = 0.1), and finally (iv) high migration (J_B_ = 0.1) with a high proliferation rate (d_B_ = 0.1).

For case (i), with both physiological rates, a linear fit was sufficient to describe the time dependence of the radius of gyration (Figure 6B; R^2^ value of 0.997, Tables S1 and S2). Using the slope of the linear fit, we were able to determine the diffusive rate of the spreading EC cluster to be 281 μm^2^/hr. Taking the diameter of the cluster to be approximated by 4 σ (which contains 95% of the cells for a Gaussian distribution) allows for direct comparisons with cluster diameters from simulations and experiments that were obtained by binarizing and thresholding images. In this case, we obtain a cluster with diameter 633 μm after 96 hrs. It is to be noted that this diameter is for a single cluster, in the absence of other competing cell types, is much larger than the measured values from experiments and simulations of about 350 μm. This suggests that the leading diffusive front region of the cluster with low cell densities is potentially outcompeted by the surrounding cell type with only the inner core region surviving as a cluster in the competitive environment. Indeed, the inner core region for these physiological parameter values is roughly half the diameter of the full cluster (Figure 6B) yielding a radius of about 320 μm, consistent with measured cluster diameters in the experiment.

For high (10 times greater) migration rates and physiological proliferation rate, we find that a linear fit can explain the radius of gyration time dependence (R^2^ = 1). As expected, the diffusive constant is roughly 10 times greater than the physiological conditions’ at 2931 μm^2^/hr. The measured cluster diameter at 96 hrs is also much larger at 2108 μm (Figure 6C). Moreover, we observed that the growing cluster does not retain its dense EC inner core, but rather the whole cluster is completely composed of fractional cell densities (lattice sites containing less than one EC). Consequently, while the increase in migration does ultimately produces a larger cluster, the lack of the core as visualized in the cluster by the absence of a bright red area (Figure 6C inset) indicates there may not be any distinct clusters in the competitive environment with multiple cell types. This is consistent with the lack of distinct patterning reported at high migration rates in our simulations (Figure 3B).

Next, we explored physiologically typical migration rates with high (4 times greater) proliferation rates. Here we noticed that the radius of gyration time dependence (Figure 6D) could not be fit by linear function and was better fit by a quadradic (R^2^ = 0.999) (Tables S3 and S4). From this quadratic fit we could extract the ballistic growth speed of the cluster, which we found to be 15.3 μm/hr. The ballistically growing cluster was observed to display both a smaller leading edge composed of fractional ECs and a larger growing inner core composed of saturated EC lattice sites (bright red area within the cluster consisting of 10 ECs per lattice site Figure 6D inset). This cluster was calculated to have a diameter of 1339 μm (compare to diameter of 633 μm in the physiological case) demonstrating that proliferation alone can drive the growth of the cluster significantly. The presence of a dominant inner core region suggests that high proliferation can also lead to clusters that are distinct in situations where both cell types are present.

Last, for both high migration and high proliferation rates, both linear and quadradic regimes (Figure 6E) were observed, indicating that a transition occurs in the growth dynamics of the cluster, specifically from linear diffusive spread to ballistic growth. We estimated the transition time point as the time at which the R^2^ value of the linear fit falls below 0.99, which in this case was at 49 hrs (Table S5). A quadratic fit on the remainder of the radius of gyration curve resulted in an R^2^ value of 0.9996 (Tables S3 and S4). This suggests that high proliferation rates can act as an additional driving force leading to an increasing in cluster size, while migration can synergistically enhance the ballistic growth phase. This is apparent when comparing the cluster size at various time points. At 49 hrs, just after the diffusive growth state, the cluster size is 1647 μm, while at 96 hrs the cluster is at 3862 μm.

### 3.5. Contact-inhibited cell migration

**Figure 7.**
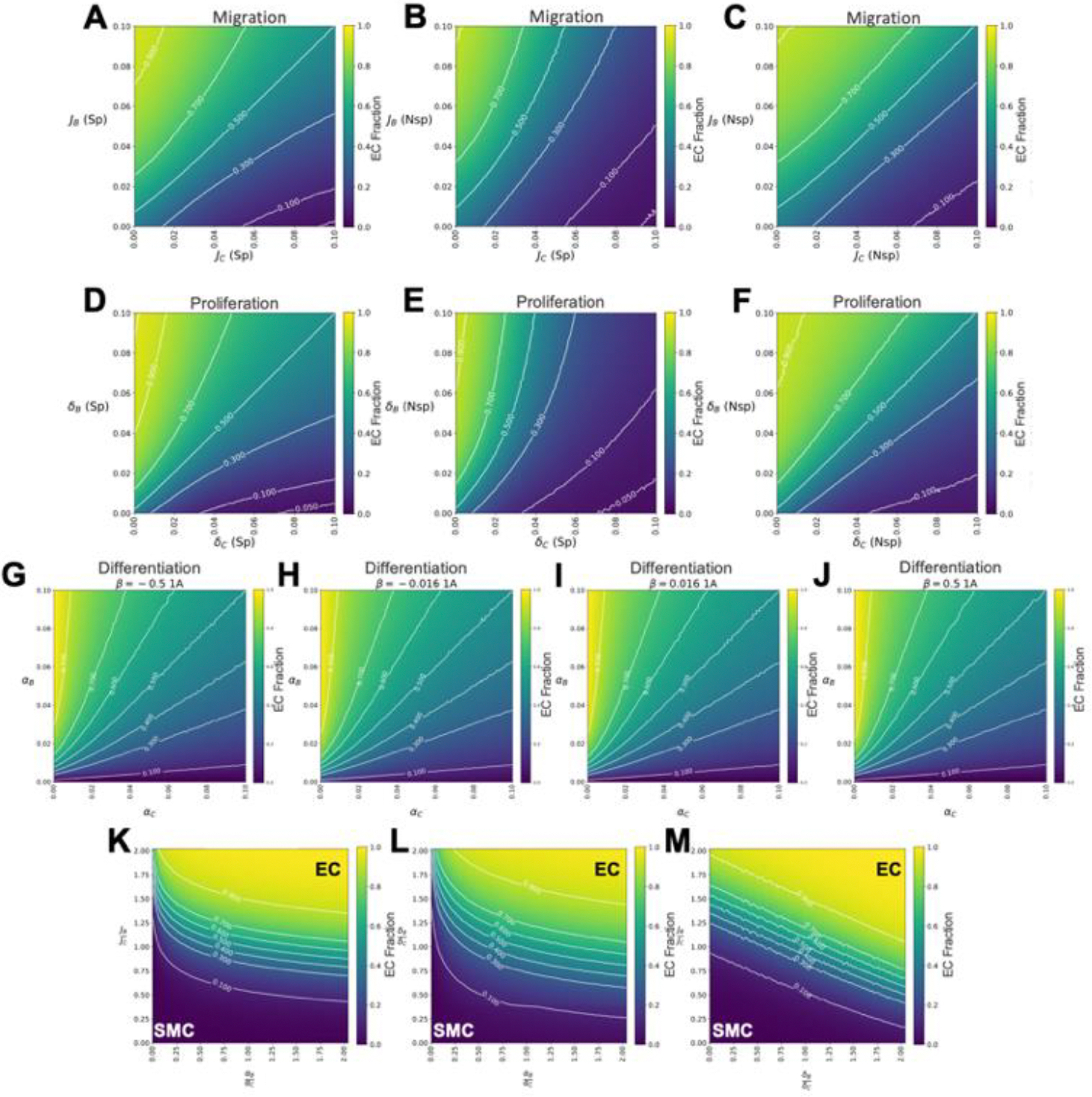
Cell migration, proliferation, and differentiation modified by cell-cell interactions. A–C) Parameter sweeps of migration rates modified under different types of interactions [specific adhesions (Sp), and nonspecific adhesions (Nsp)]. Here migration rates are varied while proliferation and differentiation rates are held constant under three EC and SMC sensing combinations: A) Specific-Specific B) Nonspecific-Specific and C) Nonspecific-Nonspecific, respectfully. D–F) Similar parameter sweeps were explored for proliferation rates under the same three sensing combinations: D) Specific-Specific, E) Nonpecific-Specific, and F) Nonspecific-Nonspecific. G–J) Parameter sweeps of differentiation rates modified by different paracrine signaling (see supplementary Figure S3), while migration and proliferation rates were held constant. A total of 16 combinations were explored under four different *β* values (*β* = −0.05, −0.016, 0.016, and 0.05). Displayed here is the 1A combination (corresponding to the differentiation of ECs and SMCs influenced by the presence of the surrounding ECs) for all four *β* values G) *β* = −0.05, H) *β* = −0.016, I) *β* = 0.016, and J) *β* = 0.05. K) EC fractions for relative ratios of migration (*J*_B_/*J*_C_) and differentiation (α_B_/α_C_). Here the migration of SMCs (J_C_) is set to displacements of 14 μm over a hr, while EC migration (J_B_) is varied between no motion and twice the SMC migration. For differentiation, SMC differentiation (α_C_) is set at one cell differentiating every 62.5 hrs and EC differentiation (α_B_) is varied between cells differentiating at twice the rate to cells that never differentiate. L) Phase diagram for relative ratios of proliferation (δ_B_/δ_C_) and differentiation (α_B_/α_C_). Here SMC proliferation (δ_C_) is set equal a cell dividing every 40 hrs, and ECs proliferation is varied between no cell divisions to twice the rate of SMCs. M) Phase diagram for ratios of migration and proliferation. Migration and proliferation ratios are the same as mentioned prior.

In typical cell culture systems, cells sense and often adhere to their neighboring cells via integrin and cadherin binding proteins. These types of cellular interactions have been shown to regulate the cell’s migration through contact-mediated inhibition [50]. Therefore, we incorporated these effects into our model by appropriately modifying the migration rates for ECs (B cells) and SMCs (C cells) to allow for specific (Sp) and nonspecific (Nsp) cell adhesions (Eqs (5) and (6)). We examined the three possible distinct combinations of these dependencies: B_Sp_-C_Sp_, B_Nsp_-C_Sp_ (same as its inverse), and B_Nsp_-C_Nsp_ (Figure 2D). The results show that when both ECs and SMCs display specific adhesions (such as B_Sp_-C_Sp_) the zone of co-emergence, defined as EC fractions between 0.3 and 0.7, is blurred and broadened (Figure 7A) compared to the control case with no contact-inhibition (Figure 3A). At higher migration rates, the contour lines exhibit a slight concave curve due to these cells’ migration being significantly slowed by their cell-to-cell adhesions (Figure 7A). Indeed, when one cell type displays specific adhesions and the other displays nonspecific adhesions (B_Nsp_-C_Sp_), the line of co-emergence curves towards the nonspecific adhering cell type (Figure 7B), indicating that the non-specifically adhering cells are more constrained. Last, when both cell types display nonspecific cell adhesions (B_Nsp_-C_Nsp_) they are similarly slowed indicated by the resulting linear contour lines (Figure 7C). These trends are also seen in the corresponding asymmetry plots (Figure S6A–C). The EC density variance (Figure S6G–I) shows fairly distinct micropatterning is present within the zone of co-emergence in all cases except when both cell types have high migration values. The patterning is dramatically more distinct in the zone of co-emergence when one cell type displays specific adhesions and the other displays nonspecific adhesions (Figure S6H). Thus, certain combinations of sensing mechanisms can actually increase the robustness of the patterning.

### 3.6. Cell density dependent proliferation

It is well-known that highly confluent monolayers of many cell types will stop proliferating due to contact inhibition, and a corresponding increase in mechanical constraints [41]. We implement contact-inhibited cell growth in our model by modifying the cell proliferation rates based on the local cell density with either specific or nonspecific sensing, Eqs (7) and (8). The behavior of the EC (B cell) fraction over the explored proliferation rate parameter space was examined for three distinct combinations of specific and nonspecific conditions, B_Sp_-C_Sp_, B_Nsp_-C_Sp_, and B_Nsp_-C_Nsp_ (Figure 7D–F), just as explored in cell migration. Adding specific and non-specific sensing to varying proliferation rates was qualitatively similar to those obtained from varying migration, although with stronger affects. When both cells display specific sensing, the zone of co-emergence, defined as the area between 0.3 to 0.7 EC fraction, is again blurred and broadened (Figure 7D) compared to the unconstrained control (Figure 4A). Additionally, when one cell type displays nonspecific sensing and the other displays specific sensing, the line of co-emergence curves towards the nonspecific sensing cell type (Figure 7E). Last, when both cell types display nonspecific sensing and proliferation is similarly slowed down, the contour lines straighten (Figure 7F). These trends can also be seen in the cell asymmetry plots (Figure S6D–F). Like the case with migration, the EC density variance (Figure S6J–L) again shows distinct micropatterning is present within the zone of co-emergence in all cases (except when both cell types have high proliferation values), and particularly so when one cell type displays specific adhesions and the other displays nonspecific adhesions (Figure S6K).

### 3.7. Cell differentiation with adjacent cell signaling

Moreover, we looked at how the patterning depends on cell differentiation rates, α, that are affected by the signaling from neighboring cells, i.e., paracrine signaling. Our model incorporates both same cell-directed differentiation and alternate cell-directed differentiation (see Eq (9) and Figure 2E). This sensing combination and the degree of amplification or suppression is controlled by the magnitude and sign of *β* (Figure S3). We explored different combinations of paracrine signaling dependence and found that, regardless of the sign and magnitude of *β*, the EC fraction, over the explored differentiation rate parameter space, was largely unaffected with no significant difference in the formation of micropatterning (Figure 7G–J). This is consistent with the lack of impact that differentiation rates ultimately have on pattern formation as long as the differentiation rates of EC/SMC cell types are similar. For full results see Supplementary Figure S7 and corresponding asymmetry and variance plots (Figure S8)

### 3.8. Relative sensitivity to different parameters

To compare the relative influence of migration, proliferation, and differentiation on micropattern formation, we explored the EC (B cell) fraction as a function of physiologically relevant ratios of these rates between the different cell types (Figure 7K–M). We first explored the EC fraction as a function of the relative migration rates, *J*_*B*_*/J*_*C,*_ and relative differentiation rates, α_B_/α_C_ (Figure 7K). For physiologically relevant scenarios with ratios between 0.5 to 2, the EC fraction contours run approximately parallel to the α_B_/α_C_ axis, thus indicating that the emergence of micropatterns is much more sensitive to the ratios of migration rates. Similar results are obtained when examining the proliferation rate ratio, δ_*B*_*/δ*_*C*_, versus the differentiation rate ratio α_B_/α_C_ (Figure 7L), with the EC fractions dependent on the proliferation more significantly than differentiation. Then, looking at the relative effects of ratios of migration and ratios of proliferation rates for ECs and SMCs (Figure 7M) reveals comparable sensitivity to relative changes in both migration and proliferation rates (Figure S9). The slopes of the EC fraction contour lines are just around ~0.5 indicating a slightly higher sensitivity to the migration rates as compared to the proliferation rates.

## 4. Discussion and conclusions

Here, we presented an on-lattice stochastic population-based model that qualitatively and quantitatively reproduces the observed 2D micropatterns that emerge during the co-differentiation of ECs and SMCs from VPCs. Our model enables the spatial visualization of this dynamic system over time and examines the effects that various biological processes on cellular micropattern development. Specifically, our computational model explored the roles that cell migration, proliferation, and differentiation, as well as contact inhibition and adjacent cell paracrine signaling have on pattern formation.

Our main finding is that cell migration and proliferation are the key factors driving 2D micropattern formation. While VPC differentiation into ECs and SMCs is required, it is not a predominant driving force in pattern formation. This finding supports an assembly mechanism for the emergence of micropatterns consistent with literature demonstrating that migration [61] and proliferation [38] can similarly drive pattern formation. These two biological processes are also the two main driving forces behind wound healing [35,62], during which, proper organization and patterning of multi-cellular tissues must be executed flawlessly. Furthermore, while some stem cell populations are present within the skin’s stem cell niche [63], their role is mainly to supply differentiated cells that will migrate into the wound and proliferate.

The emergence of distinct micropatterns is achieved over a broad area of parameter space, termed the “zone of co-emergence”, where EC fractions are roughly between 0.30–0.70. Coupled with our finding that the inclusion of neighbor cell sensing mechanisms impacts the shape of the zone of co-emergence, this proved to be a strong predictor for the development of micropatterns that may be application to other biological systems with implications in directing in-vitro 3D organ morphogenesis.

While cell proliferation and migration rates are the primary driving forces that enable micropatterning, the specific rates and the presence of contact inhibition predict the degree of micropattern development and the length scales of the patterns. Our work on single cluster growth dynamics suggests that the cluster sizes are set by how far the inner core regions, containing high cell densities, can expand and occupy space before being outcompeted by the other cell type. While contact inhibition mitigation of migration and proliferation rates can become important, fewer differences are observed between homotypic and nonspecific sensing mechanisms at low migration and proliferation rates. As rates increase, we see a broad and symmetrical expansion of conditions enabling micropattern development. This is true when ECs and SMCs are both sensing homotypically and nonspecifically. However, when one cell type is sensing homotypically and the other is sensing nonspecifically, there is a reduction in the range of conditions that enable micropattern development accompanied by a significant increase in the distinctness of patterning. This suggests that sensing can help increase the robustness of patterning.

A limitation of our model is the lack of single cell specificity, such as cell shape and polarity, which are important when modeling directional propulsion and cell-to-cell adhesions [64,65]. This level of single cell modeling, at high resolutions, would be excessive for our population-based questions. However, our modeling framework does allow for the modification of our lattice site dimensions, which serves to increase or decrease the overall resolution of our system, within reason, by directly affecting the number of cells that can inhabit a single lattice site. Our modeling framework could also be applicable to study the development of other cells or tissues like the emergence of keratinocytes during differentiation, wound healing, and normal skin repair [66] or the precise staggered patterning of R8 photoreceptor precursors with accessory cells in the neural epithelium of *Drosophila* eyes [67].

We have explored a range of migration, proliferation, and differentiation values to assess the impact each has on the emergence of multicellular micropatterns within developing vascular tissue and found that patterning is dominated by migration and proliferation under physiologically relevant conditions for VPCs. Our work strongly suggests that, even in the absence of any specific mechanisms that drive segregation, like chemotaxis, 2D micropatterning can emerge as long as cellular fractions are maintained within 0.30–0.70, with cluster sizes being set by the growth rate of single cluster inner cores. Our results suggest that even though micropatterning can occur in the absence of sensing, the presence of such mechanisms greatly increases the robustness of patterning, which could be critical to fidelity in tissue development in the naturally noisy and heterogeneous *in vivo* setting.

## Supplementary Material

supplemental videos

Supplemental Tables and Figures

## Figures and Tables

**Table 1.** Parameter values used in model. Variables are fixed at these experimentally obtained values for migration, proliferation, and/or differentiation while explicitly varying others. In-silico unit time step corresponds to 1 hour and unit length corresponds to 79 μm (the lattice size).

Variable	Denotation	Experimentally obtained values	Corresponding simulation value(s)	Parameter sweep	References

Diffusion constant	J_θ_	14 μm^2^/hr	0.0079	0–0.1; 0.001 step size	Supplemental video V1 (37–38)
Proliferation rate	δ_θ_	~40 hr	0.025	0–0.1; 0.001 step size	(39–41)
Differentiation rate	α_θ_	~62.5 hr	0.016	0–0.1; 0.001 step size	(19,20)
Stochastic noise	S_θ_	A loss or gain of up to 1 cell per lattice site is incorporated every 10 hrs	± 0.01	N/A	NA
Paracrine signal strength	β	amplification of differentiation rates by 0.5–1.5	± 0.5, ± 0.016, 0	N/A	NA
