# Supplementary material for: Migration and proliferation drive the emergence of patterns in co-cultures of differentiating vascular progenitor cells
Source: Math Biosci Eng. Author manuscript; Available in PMC 2024 Nov 12. (PMC11556463; doi:10.3934/mbe.2024295)
Supplement: Supplemental Tables and Figures [file NIHMS2030393-supplement-Supplemental_Tables_and_Figures.pdf]

Mathematical Biosciences and Engineering

MBE, 21(8): 6731–6757.
DOI: 10.3934/mbe.2024295
Received: 07 May 2024
Revised: 27 June 2024
Accepted: 16 July 2024
Published: 01 August 2024

https://www.aimspress.com/journal/MBE

Research article

Migration and proliferation drive the emergence of patterns in co-cultures of differentiating vascular progenitor cells

Jose E. Zamora Alvarado[1,2], Kara E. McCloskey[1,2] and Ajay Gopinathan[2,3,*]

[1] School of Engineering, University of California Merced, Merced, CA 95343, USA
[2] Graduate Program in Materials and Biomaterials Science and Engineering, University of California Merced, Merced, CA 95343, USA
[3] Department of Physics, University of California Merced, Merced, CA 95343, USA

* **Correspondence:** Email: agopinathan@ucmerced.edu.

Supplementary

Table S1. Linear fit equations.

	$d_B = 0.001$	$d_B = 0.01$	$d_B = 0.025$	$d_B = 0.03$	$d_B = 0.05$	$d_B = 0.079$	$d_B = 0.1$
$J_B = 0.001$	Y = 19.84x -146.12	Y = 21.26x -165.53	Y = 17.64x - 43.44	Y = 16.66x - 31.43	Y = 14.87x -19.04	-	-
$J_B = 0.0079$	Y = 286.79x - 1956.65	Y = 283.86x - 1940.75	Y = 281.36x - 1986.97	Y = 248.59x - 260.54	Y = 224.94x - 959.26	Y = 106.93x - 116.78	-
$J_B = 0.01$	Y = 371.96x - 2246.09	Y = 365.01x - 2126.33	Y = 362.36x - 2166.01	Y = 355.47x - 2117.49	Y = 337.73x - 1807.71	Y = 144.67x - 169.30	-
$J_B = 0.03$	Y = 1118.94x - 2729.90	Y = 1116.90x - 2960.47	Y = 1098.93x - 2816.26	Y = 1092.09x - 2744.39	Y = 1066.05x - 2642.59	Y = 1049.67x - 2865.79	Y = 1014.87x - 2619.39
$J_B = 0.05$	Y = 1870.09x - 3139.01	Y = 1859.48x - 3232.19	Y = 1836.85x - 3301.56	Y = 1816.78x - 3036.47	Y = 1782.94x - 3262.71	Y = 1725.46x - 2975.61	Y = 1710.48x - 3217.93
$J_B = 0.079$	Y = 2999.64x - 3618.62	Y = 2974.24x - 3769.00	Y = 2931.58x - 3576.58	Y = 2902.39x - 3457.22	Y = 2852.90x - 3577.29	Y = 2773.15x - 3521.62	Y = 2729.22x - 3531.51
$J_B = 0.1$	Y = 3732.18x - 3770.78	Y = 3705.19x - 3935.06	Y = 3644.27x - 3569.18	Y = 3639.61x - 3949.63	Y = 3561.01x - 3788.22	Y = 3472.11x - 4032.90	Y = 3401.06x - 3838.41

Table S2. R^2 fit values for linear equations.

	$d_B = 0.001$	$d_B = 0.01$	$d_B = 0.025$	$d_B = 0.03$	$d_B = 0.05$	$d_B = 0.079$	$d_B = 0.1$
$J_B = 0.001$	0.987	0.988	0.993	0.995	0.998	NaN	NaN
$J_B = 0.0079$	0.998	0.998	0.997	0.994	0.991	1	NaN
$J_B = 0.01$	0.999	0.999	0.999	0.999	0.996	0.999	NaN
$J_B = 0.03$	1	1	1	1	1	1	0.999
$J_B = 0.05$	1	1	1	1	1	1	1
$J_B = 0.079$	1	1	1	1	1	1	1
$J_B = 0.1$	1	1	1	1	1	1	1

Table S3. Quadratic fit equations.

	$d_B = 0.001$	$d_B = 0.01$	$d_B = 0.025$	$d_B = 0.03$	$d_B = 0.05$	$d_B = 0.079$	$d_B = 0.1$
$J_B = 0.001$	-	-	$Y = 0.38x^2 + 2.16x + 125.86$	$Y = 0.51x^2 - 1.29x + 157.69$	$Y = 0.91x^2 - 1.45x + 33.32$	$Y = 2.21x^2 - 40.24x + 367.39$	$Y = 3.01x^2 - 50.67x + 359.71$
$J_B = 0.0079$	-	-	-	$Y = 3.20x^2 + 263.59x - 1944.62$	$Y = 7.70x^2 - 431.20x + 13964.86$	$Y = 11.18x^2 - 245.55x + 3268.49$	$Y = 15.25x^2 - 332.77x + 3437.50$
$J_B = 0.01$	-	-	-	-	$Y = 11.68x^2 - 931.27x + 32528.30$	$Y = 13.90x^2 - 319.12x + 4407.44$	$Y = 18.70x^2 - 409.63x + 4193.76$
$J_B = 0.03$	-	-	-	-	-	$Y = 53.97x^2 - 3495.21x + 86527.69$	$Y = 62.63x^2 - 2958.44x + 57364.95$
$J_B = 0.05$	-	-	-	-	-	$Y = 101.51x^2 - 8466.75x + 240123.21$	$Y = 108.78x^2 - 6214.86x + 131037.29$
$J_B = 0.079$	-	-	-	-	-	$Y = 199.59x^2 - 21049.61x + 691693.12$	$Y = 185.83x^2 - 12837.28x + 300726.96$
$J_B = 0.1$	-	-	-	-	-	$Y = 279.16x^2 - 32513.41x + 1138285.27$	$Y = 240.13x^2 - 17980.69x + 445481.52$

Table S4. R2 fit values for quadratic equations.

	$d_B = 0.001$	$d_B = 0.01$	$d_B = 0.025$	$d_B = 0.03$	$d_B = 0.05$	$d_B = 0.079$	$d_B = 0.1$
$J_B = 0.001$	-	-	1.0000	0.9999	0.9995	0.9975	0.9978
$J_B = 0.0079$	-	-	-	0.9998	0.9995	0.9985	0.9988
$J_B = 0.01$	-	-	-	-	0.9999	0.9987	0.9989
$J_B = 0.03$	-	-	-	-	-	0.9999	0.9999
$J_B = 0.05$	-	-	-	-	-	0.9997	0.9998
$J_B = 0.079$	-	-	-	-	-	0.9993	0.9996
$J_B = 0.1$	-	-	-	-	-	0.9991	0.9994

Table S5. Time-point transitions (in hrs) from linear to quadratic domains.

	$d_B = 0.001$	$d_B = 0.01$	$d_B = 0.025$	$d_B = 0.03$	$d_B = 0.05$	$d_B = 0.079$	$d_B = 0.1$
$J_B = 0.001$	-	-	26	12	8	2	1
$J_B = 0.0079$	-	-	-	18	39	3	1
$J_B = 0.01$	-	-	-	-	63	4	1
$J_B = 0.03$	-	-	-	-	-	50	35
$J_B = 0.05$	-	-	-	-	-	59	42
$J_B = 0.079$	-	-	-	-	-	66	49
$J_B = 0.1$	-	-	-	-	-	70	51

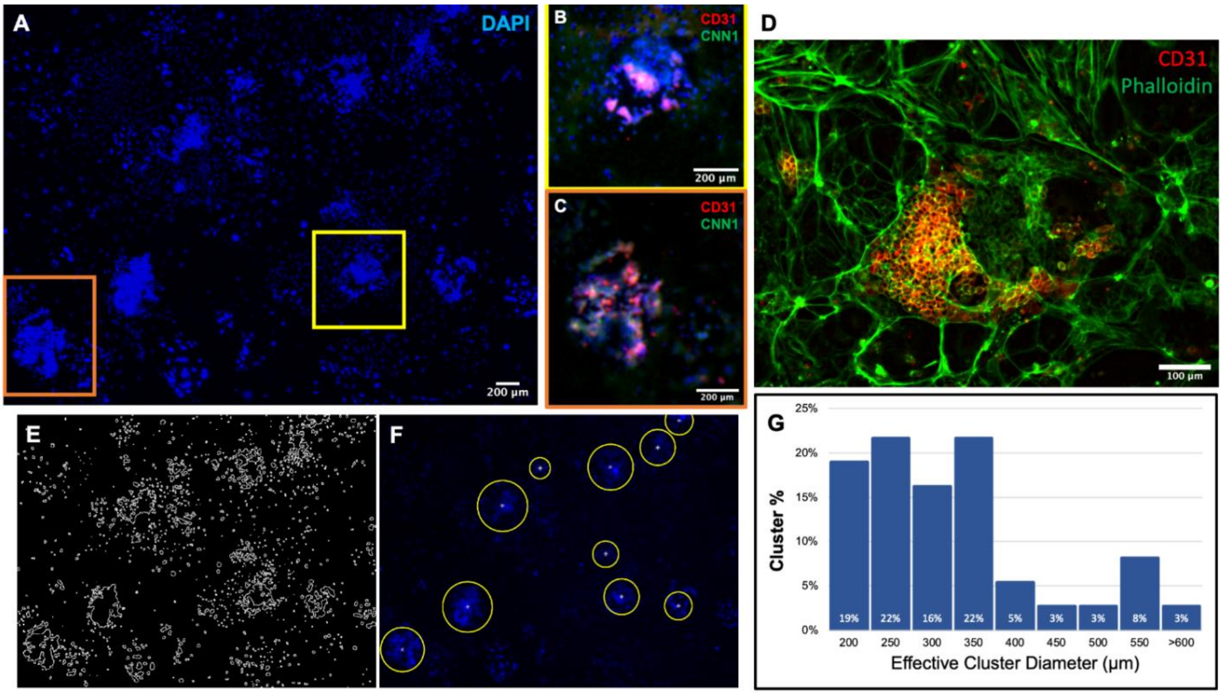

Figure S1. EC clusters emerge during differentiation. A) Image taken at low magnification captures wide distribution of cellular nuclear stain, DAPI. Nuclear staining allowed for cluster analysis by capturing areas of dense cell clusters. B and C) Cropped images of clusters show, faint, CD31 + ECs (red), CNN1 + SMCs (green), and DAPI expression. Images show the CD31 + ECs mostly present within the center of dense DAPI clusters, while CNN1 + SMCs are mostly found on the periphery of these clusters. D) A cell cluster stained for CD31 + ECs counterstained with phalloidin (green). Phalloidin binds to F-actin, a common cytoskeleton protein. E–G) A custom MATLAB script was used to identify EC clusters and determine the effective cluster diameters distribution from the DAPI stain. E) Shows the binary outline of the DAPI stain after some image processing. D) Dense clusters are then identified by thresholding their area, shown here encased by yellow circles with a white star identifying their center. G) Graph of the effective cluster diameter distribution, with a bin size of 50 μm. Here the mean effective cluster diameter was calculated to be 340 ± 110 μm taken from a total of 4 experimental cultures.

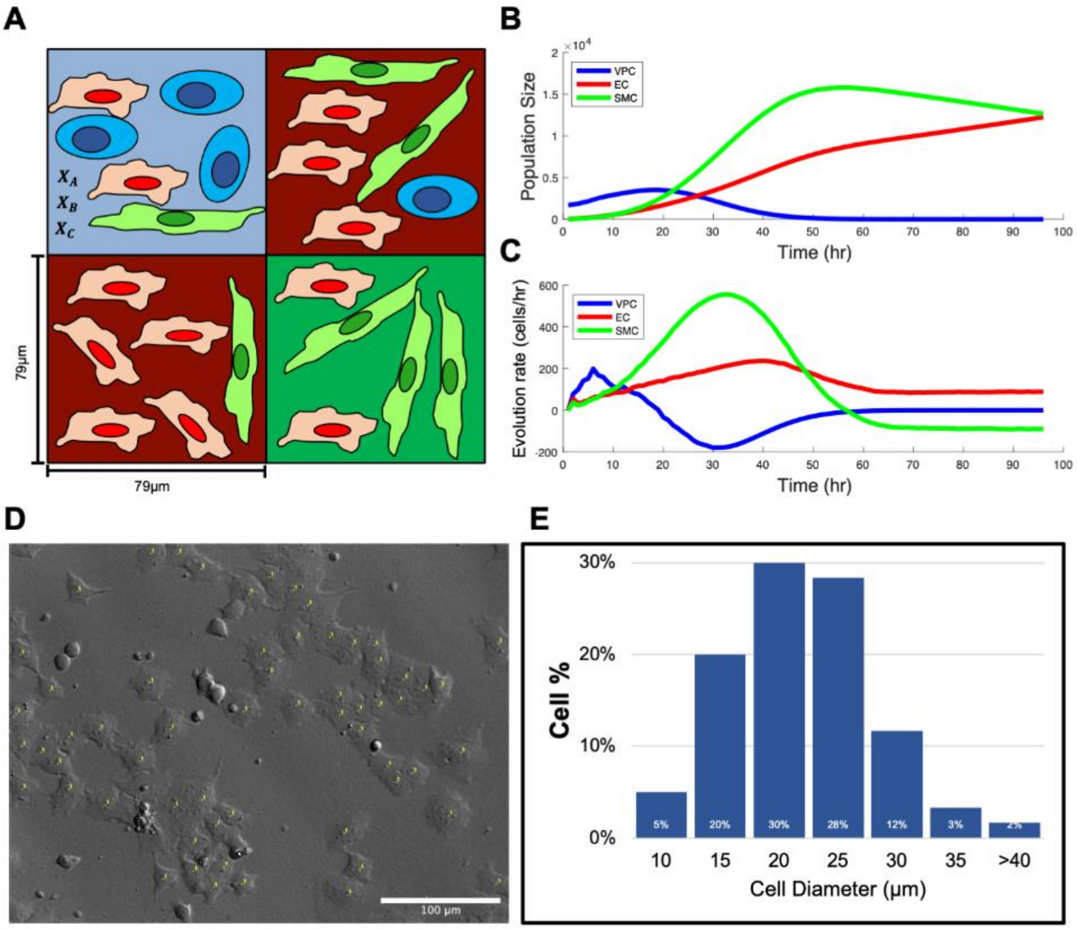

Figure S2. Relating simulations to experiments A) Schematic of a 2x2 lattice, where each lattice site has a length 79 μm and can house a maximum of 10 cells. As the simulation evolves in time, the cell distributions are visualized by their RGB color assignment (red-EC, green-SMC, blue-VPC). While the simulation is running B) the total population size and C) evolution/devolution rates, a relative measurement of the number of cells added or removed between time steps, are tracked for all three cell types. In this example, we assume paracrine signaling with a β value equal to 0.6. Additionally, migration, J_θ, was the same for all cell types at 0.0078 (corresponding to 14 μm/hr), the proliferation, δ_θ, was set to 0.083 (12 hrs) for VPCs and 0.059 (17 hrs) for ECs and 0.045 (22 hrs) SMCs. Lastly, the differentiation rate, α_θ, set to 0.016 (62.5 hrs) and 0.01 (100 hrs) for EC and SMC, respectively. Note: These are same conditions as supplementary Video S2. B) We observed the VPC population quickly falls to zero in response to VPC differentiation, i.e., VPCs turn into ECs and SMCs. C) Examining the evolution/devolution rates reveals the point at which these cells reach steady state, here occurring after 60 hrs. D) Image of differentiating VPCs taken from time-lapse video (video S1). E) Cell diameter distribution from D, with a bin size of 5 μm.

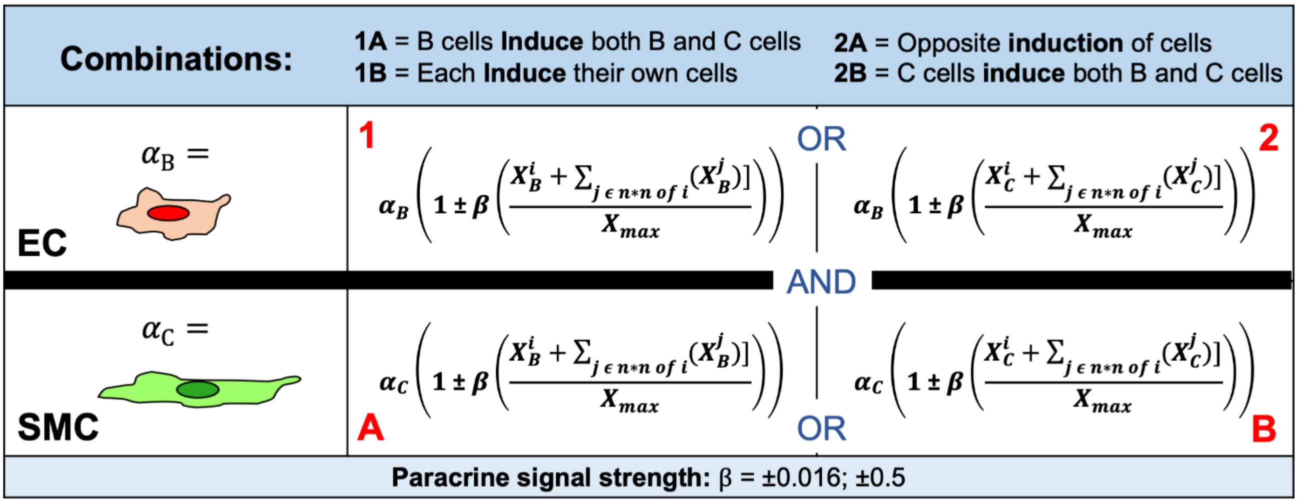

Figure S3. Differentiation equations regulating cell-directed differentiation and alternate cell-directed differentiation. Shown here are the combinations of equations that regulate same cell-directed differentiation and alternate cell-directed differentiation as well as other methods of sensing. Based on the exact combination, we can explore situations where cells induce either the same cell type or the opposite cell type, see combinations list for full details. Additionally, these equations depend on the β variable, defined as the paracrine signal strength, which acts to amplify or damp the sensed effect (for our purposes we explored β values equal to ± 0.5 and ± 0.016).

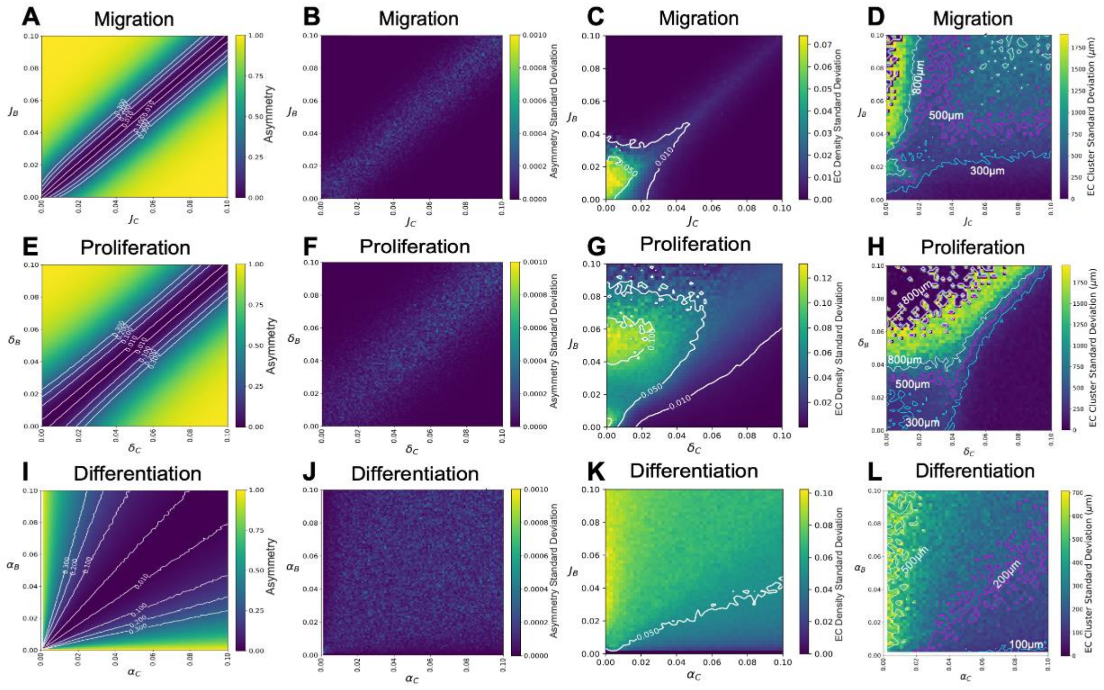

Figure S4. Cellular asymmetry plots under no sensing mechanism and standard deviation plots for Asymmetry, EC density, and EC cluster diameters. Cell asymmetry measurements reflect the relative presence of EC and SMCs. With values close to 0 indicating roughly equal densities of both cell types, while values close to 1 denoting the presence of only cell types. In conditions where VPCs are no long present after 96 hrs, these asymmetry graphs can serve as useful indicators of pattern formation. Indeed, when compared to EC fraction parameter sweeps, they tend to mirror, and even better identify, the zone of co-emergence. Presented here are the cell asymmetry parameter sweeps for A) Migration, E) Proliferation, and I) differentiation. Contour lines here reflect the 0.01, 0.1, 0.2, and 0.3 boundaries. Parameter sweeps were run 10 times for Asymmetry and EC density while only 3 times for EC cluster diameters, thus allowing us to gauge the variance over repeated simulations. Shown here are the standard deviation graphs for migration parameter sweeps of B) asymmetry, C) EC density, and D) EC cluster diameter. As well as parameter sweeps of proliferation F) asymmetry, G) EC density, and H) EC cluster diameter. Lastly, the parameter sweeps of for differentiation, J) asymmetry, K) EC density, and L) EC cluster diameter.

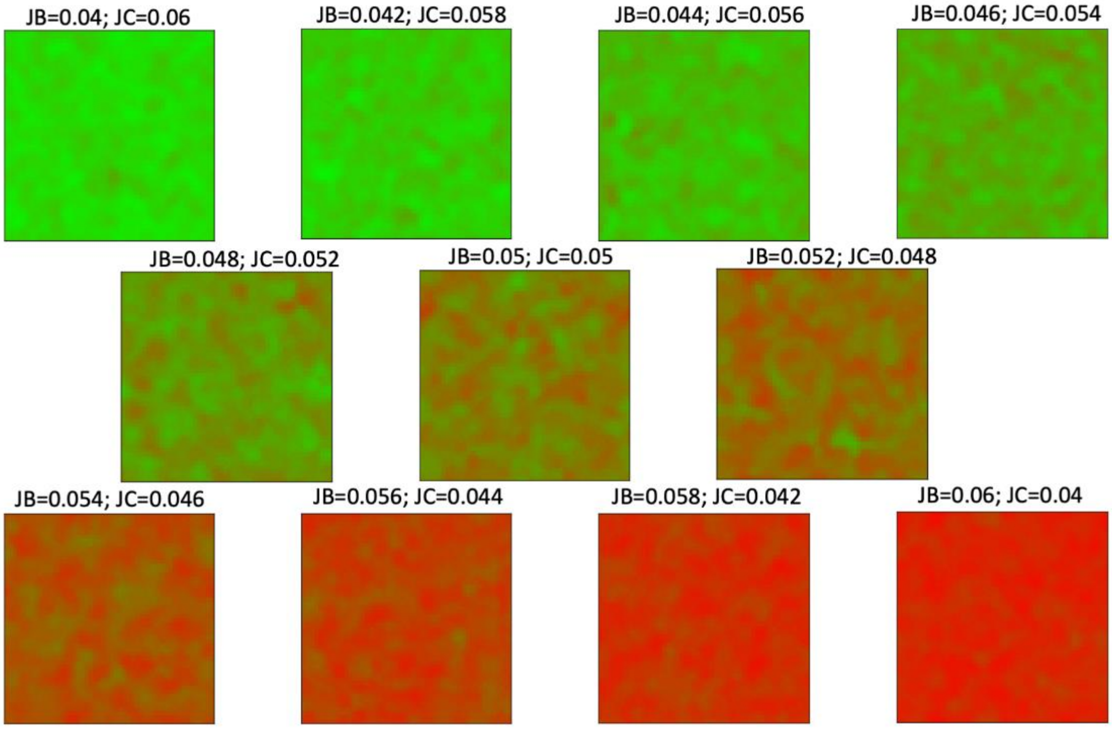

Figure S5. Cross-sectional scan of the zone of co-emergence as seen in the migration EC fraction parameter sweep. Shown here are the individual micropattern simulations for values that cross the zone of co-emergence for the non-sensing migration EC fraction parameter sweep (Figure 3A). At the point of symmetry were $J_B = 0.05$ and $J_C = 0.05$, ECs (red) and SMCs (green) clusters emerge in roughly equal quantities. By varying the migration values away from the point of symmetry a preference towards one cell type becomes apparent and benefits the faster cell type (e.g., increase J_B, favors ECs, leading to more ECs in the simulation until they eventually fill the available space leaving little space for the SMCs to fill, and vice-versa).

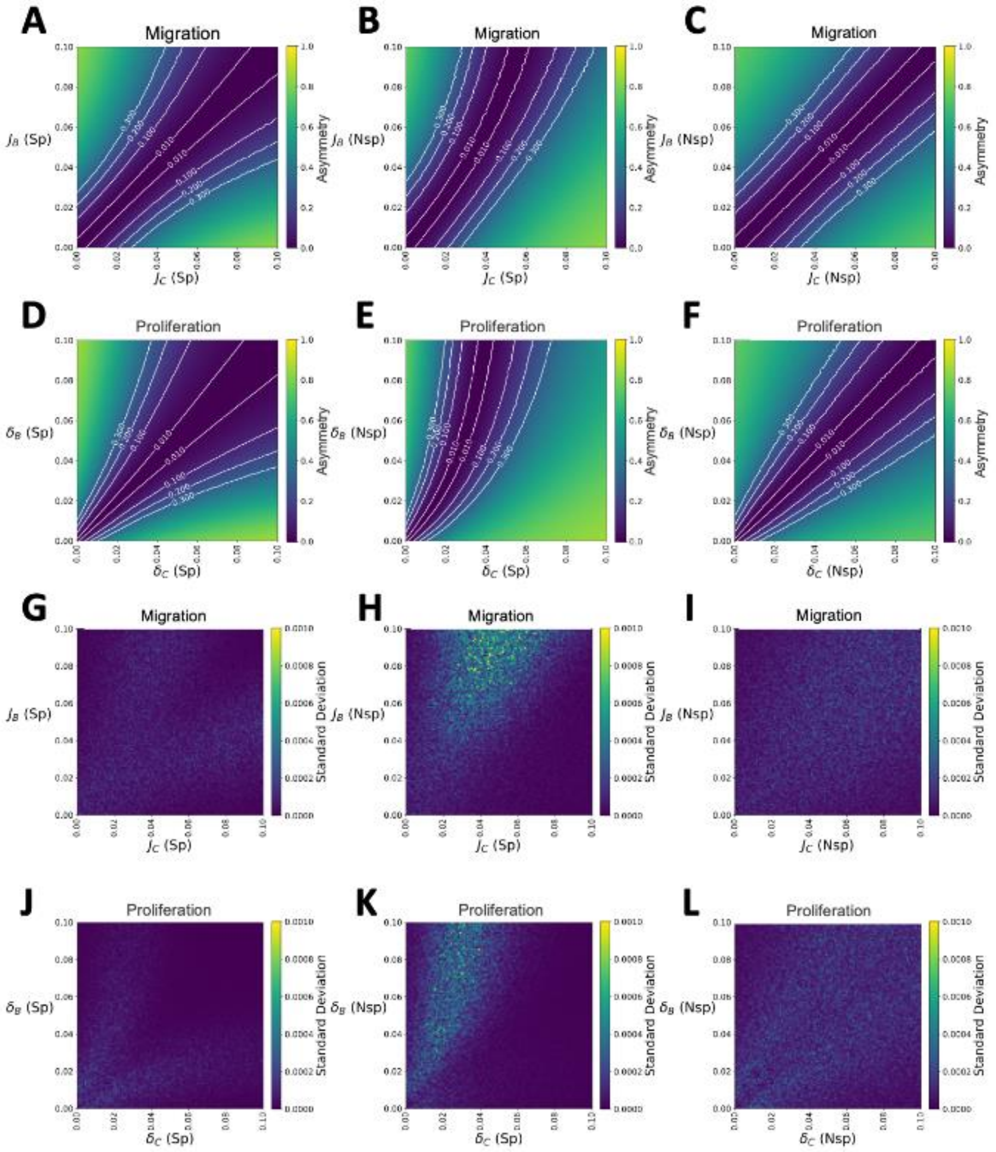

Figure S6. Cellular asymmetry plots under sensing mechanisms for migration and proliferation. Shown here are the cell asymmetry parameter sweeps for migration and proliferation under imposed sensing rules that govern the interactions VPCs, ECs, and SMCs have with each other. Explored here are specific cell adhesions (Sp) where only cells of the same identity will sense each other, and nonspecific cell adhesions (Nsp) which allows cells to sense all other cell types. The explored combinations are Specific-Specific, Nonpecific-Specific, and Nonspecific-Nonspecific for A–C) migration and D–F) proliferation, respectfully. Here values close to 0 indicate similar population densities, and the presence of patterning, while values closer to 1 denote the presence of only one cell type and thus no patterning. Simulations were run 10 times for the three conditions (Specific-Specific, Nonpecific-Specific, and Nonspecific-Nonspecific) and their standard deviations were calculated and plotted for G–I) migration and J–L) proliferation, respectfully.

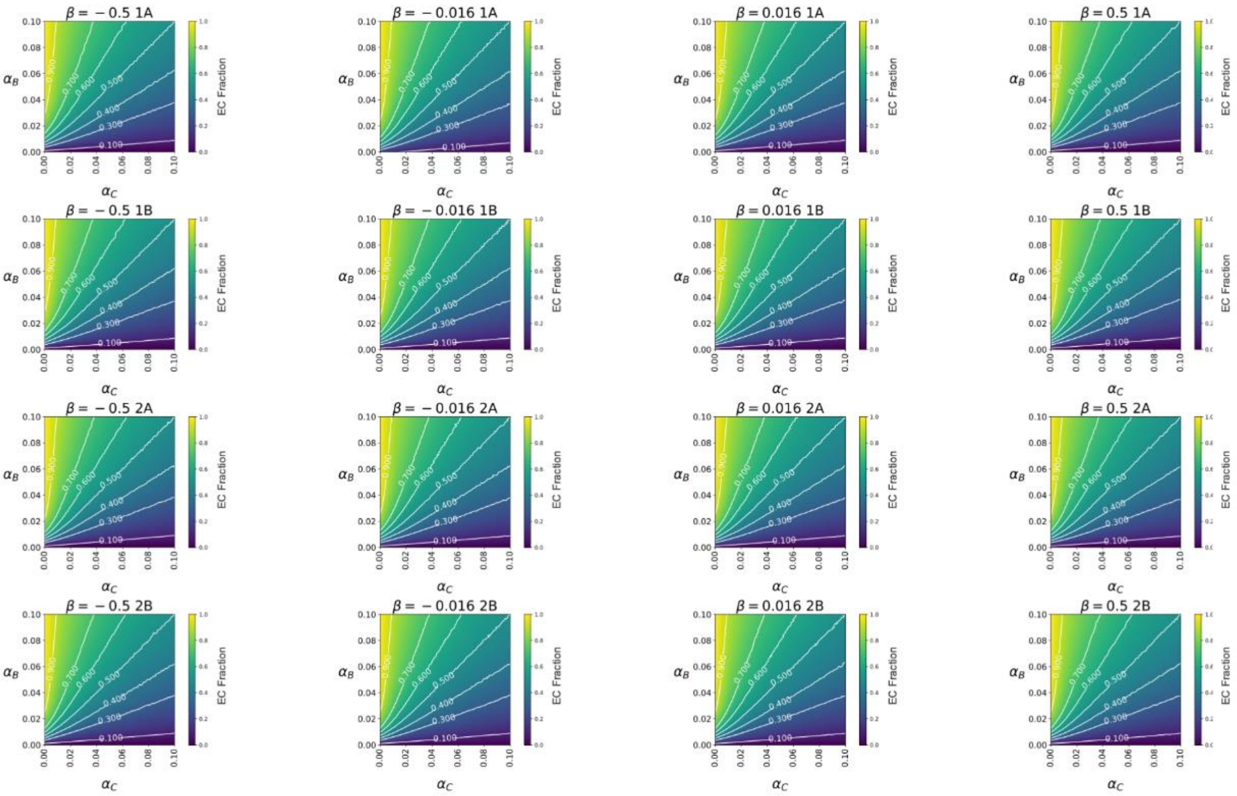

Figure S7. Total EC fraction results for differentiation under all sensing mechanisms. Shown here are all the parameter sweeps for differentiation under the four different sensing mechanisms, and under the four different β values (± 0.5 and ± 0.016). Condition 1A: ECs induce both EC and SMC differentiation. 1B: ECs induce the differentiation of ECs and SMCs induce the differentiation of SMCs, termed same cell-directed differentiation. 2A: ECs induce the differentiation of SMCs and SMCs induce the differentiation of ECs, termed alternate cell-directed differentiation. lastly 2B: SMCs induce both EC and SMC differentiation. Alas, there was no observable difference in the EC fraction plots for these different combinations.

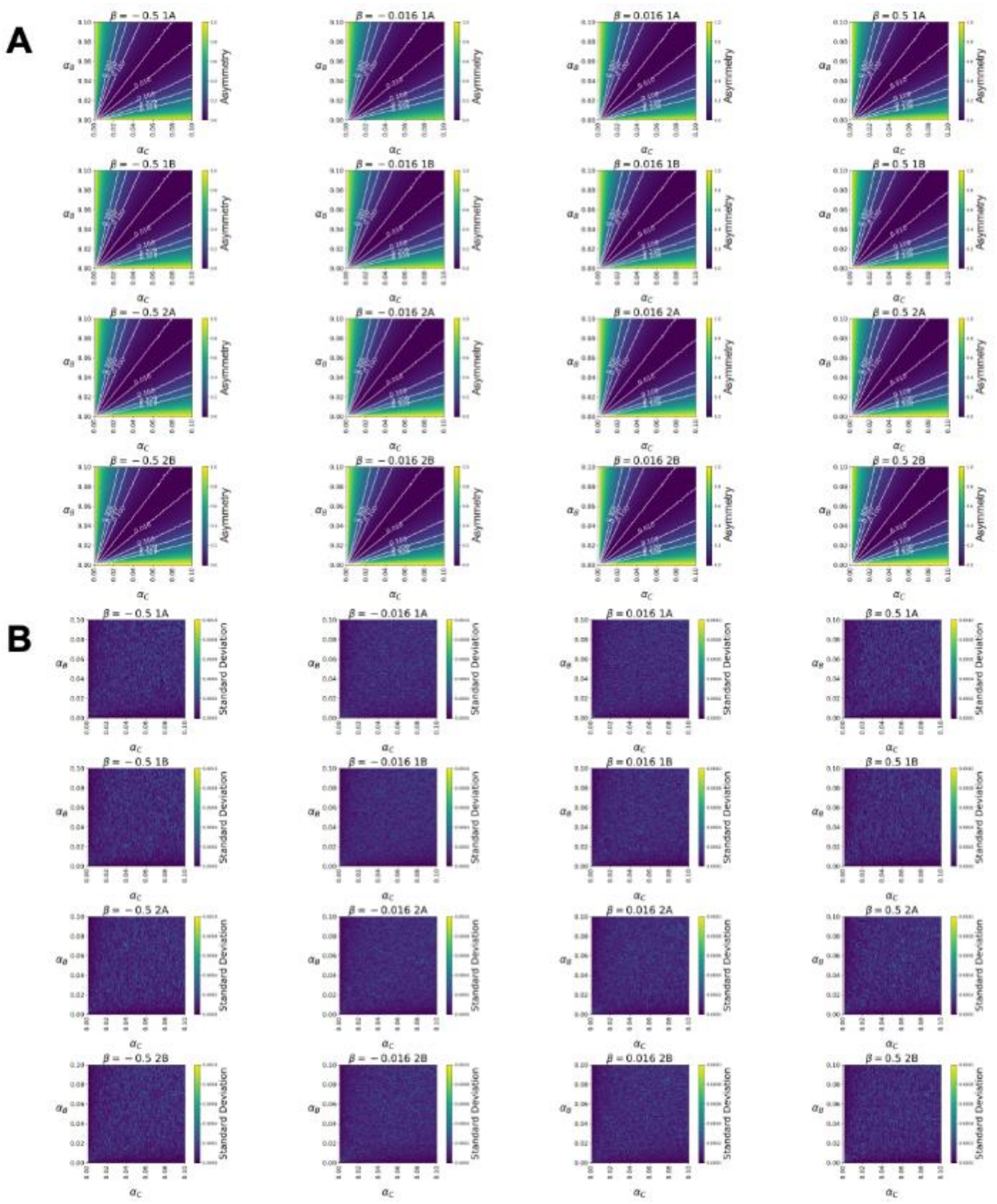

Figure S8. Cellular asymmetry plots, for differentiation under all sensing mechanisms. A) Shown here are all the cell asymmetry parameter sweeps under the four different sensing mechanisms, and under the four different β values (± 0.5 and ± 0.016). Condition 1A: ECs induce both EC and SMC differentiation. 1B: ECs induce the differentiation of ECs and SMCs induce the differentiation of SMCs. 2A: ECs induce the differentiation of SMCs and SMCs induce the differentiation of ECs. lastly 2B: is when SMCs induce both EC and SMC differentiation. Here values close to 0 indicate similar population densities, and in most cases the presence of patterning, while values closer to 1 denote the presence of only one cell type and thus no patterning. B) The corresponding standard deviations for the asymmetry parameter sweep taken from 10 replicated runs.

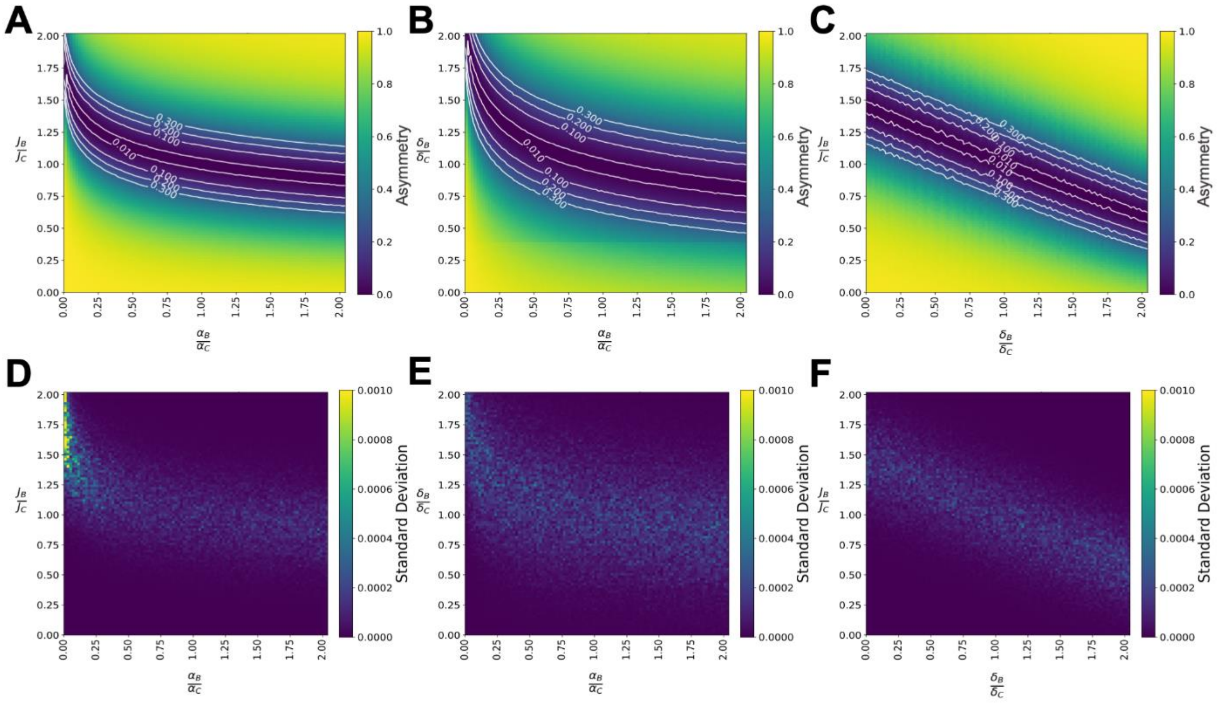

Figure S9. Multiparameter cellular asymmetry phase diagrams of micropatterning behavior. A) Cell asymmetry phase diagram for relative ratios of migration (J_B/J_C) and differentiation (α_B/α_C). Here the migration of SMCs (J_C) is fixed at 14 μm^2/hr while EC migration (J_B) is varied between no motion and twice that SMC migration. For differentiation, SMC differentiation (α_C) is fixed at a differentiation rate of 62.5 hrs and EC differentiation (α_B) is varied between cells differentiating at twice that rate to cells that never differentiate. B) Cell asymmetry phase diagram for relative ratios of proliferation (δ_B/δ_C) and differentiation (α_B/α_C). Here SMC proliferation (δ_C) is fixed at a rate of one cell division every 40 hrs, and ECs proliferation is varied between no cell divisions to twice the rate of SMCs. C) Cell asymmetry phase diagram for relative values of migration and proliferation. A–C) Values close to 0 indicate similar population densities, and the presence of patterning, while values closer to 1 denote the presence of only one cell type and thus no patterning. D–F) The standard deviations for these phase diagrams were taken from 10 replicated simulations. Shown here D) migration and differentiation, E) proliferation and differentiation, and F) migration and proliferation.

http://gopinathanlab.ucmerced.edu/datavideos.html

Video S1. Time-lapse video of differentiating VPCs. Shown here is a 24 h time-lapse video of R1 VPCs taken during day 1–2 of post differentiation supplemented with stage 2 differentiation medium.

http://gopinathanlab.ucmerced.edu/datavideos.html

Video S2. Simulation of emerging micropattern. Video of simulation shows the emergence of EC (red) and SMC (Green) micropatterning from a starting VPCs population, the starting density is equivalent to a seeding density of 10k cells/cm^2. In this example, we assume paracrine signaling, which benefits SMC differentiation (condition 1A), and with a β value equal to 0.6. Additionally, migration, J_θ, was the same for all cell types at 0.0078 (corresponding to 14 μm/hr), the proliferation, δ_θ, was set to 0.083 (12 hrs) for VPCs and 0.059 (17 hrs) for ECs and 0.045 (22 hrs) SMCs. Lastly, the differentiation rate, α_θ, set to 0.016 (62.5 hrs) and 0.01 (100 hrs) for EC and SMC, respectively.
